# Mitochondrial metabolic study guided by proteomics analysis in hepatocellular carcinoma cells surviving long-term incubation with the highest dose of sorafenib

**DOI:** 10.18632/aging.102582

**Published:** 2019-12-26

**Authors:** Jing Bai, Ziqi Liu, Jiang Liu, Saihang Zhang, Yuan Tian, Yueshan Zhang, Leiming Ren, Dezhi Kong

**Affiliations:** 1 Department of Pharmacology, Institute of Chinese Integrative Medicine, Hebei Medical University, Shijiazhuang 050017, Hebei, China; 2 Department of Pharmacy, Fourth Hospital of Hebei Medical University, Shijiazhuang 050017, Hebei, China; 3 Department of Hepatobiliary Surgery, Fourth Hospital of Hebei Medical University, Shijiazhuang 050017, Hebei, China

**Keywords:** hepatocellular carcinoma, insensitivity, mitochondria, complex I, sorafenib

## Abstract

Sorafenib is the standard first-line systemic therapy for hepatocellular carcinoma (HCC). However, the low objective response rates in clinical studies suggest the existence of certain HCC cells that are inherently insensitive to sorafenib. To understand the molecular basis of insensitivity of HCC cells to sorafenib, this study developed 3 kinds of insensitive HCC cells through exposure to various concentrations of sorafenib and performed a quantitative proteome analysis of the surviving HepG2 cells. 520 unique proteins were concentration-dependently upregulated by sorafenib. Bioinformatics-assisted analysis of 520 proteins revealed that the metabolic pathways involved in central carbon metabolism were significantly enriched, and 102 mitochondrial proteins, especially components of the electron transport chain (ETC), were incrementally upregulated in the 3 kinds of insensitive cells. Conversely, we identified a rapid holistic inhibitory effect of sorafenib on mitochondrial function by the direct targeting of the complex I-linked electron transport and the uncoupling of mitochondrial oxidative phosphorylation (OXHPOS) in HCC cells. Core metabolic reprogramming involved in a compensatory upregulation of OXHPOS combined with elevated glycolysis supports the survival of HCC cells under the highest dose of sorafenib treatment. Altogether, our work thus elaborates an ETC inhibitor and unveils the proteomic landscape of metabolic reprogramming in drug insensitivity.

## INTRODUCTION

Overall incidence and mortality rates for cancer are declining, but the incidence and mortality rates for liver cancer are increasing [1, 2] with hepatocellular carcinoma (HCC) accounting for 70%–90% of primary liver cancers [3]. The prognosis of HCC patients remains poor, and approximately 30%–40% of the patients qualify for potentially curative or locoregional therapies [4, 5], indicating a lack of effective treatment options for HCC [6]. Frequently, systemic therapy is the only option for patients with advanced HCC. Sorafenib is the first targeted drug approved by the FDA for use in HCC in 2007 and today, sorafenib is still recommended as the standard first-line systemic therapy for HCC patients with advanced disease according to the US National Comprehensive Cancer Network (NCCN) Clinical Practice Guidelines [5]. Even though treatment of advanced HCC with sorafenib prolongs median overall survival by 2–3 months [7, 8], the clinical outcome is far from satisfactory.

To improve clinical efficacy of sorafenib, considerable efforts have been made to understand the mechanisms of action and resistance and to explore the strategies for overcoming resistance to sorafenib [9, 10]. It is well known that the multikinase inhibitor sorafenib inhibits tumor cell proliferation and angiogenesis by inhibiting serine-threonine kinase RAF (B and C) and several tyrosine kinase sites including vascular endothelial growth factor receptor (VEGFR)-1, VEGFR-2, VEGFR-3, platelet-derived growth factor receptor (PDGFR)-β, Fms-related tyrosine kinase 3 ligand (FLT-3) and cytokine receptor c-Kit [11–13]. The lack of efficacy of several other tyrosine kinase inhibitors in HCC treatment suggests that the mechanisms beyond kinase inhibition contribute to higher anticancer activity of sorafenib [14]. Numerous studies aim to investigate the inhibition of mitochondrial oxidative phosphorylation (OXPHOS) as a mechanism contributing to the antiproliferative activity of sorafenib in HCC cells [15], as there has been evidence that sorafenib inhibits mitochondrial respiratory function and reduces intracellular ATP levels [16]. However, the exact mechanisms of sorafenib inhibition of OXPHOS have not been fully elucidated. In particular, studies involving resistant HCC cell lines [17], tumor tissue materials [18] or serum derived from the patients [19] revealed that sorafenib resistance is associated with the upregulation of several signaling pathways, such as Akt S473 phosphorylation [20], the mammalian target of rapamycin (mTOR) pathway [21], and epithelial-to-mesenchymal transition (EMT) [22]. The combination strategies to explore a possibility of overcoming the resistance to sorafenib remain in the early phase [23–25].

In fact we cannot ignore that in pivotal SHARP and Asia-Pacific studies, the objective tumor response rates were only 2% to 3% in sorafenib-treated patients [7, 8], suggesting the existence of a certain fraction of HCC cells that are initially insensitive to sorafenib treatment; this phenomenon is a formidable obstacle preventing improvement in the treatment efficacy. A survival pathway is not inhibited by sorafenib in the insensitive HCC cells. This point of view is supported by the studies of leukemic stem cells insensitive to inhibition by a BCR-ABL kinase inhibitor imatinib in chronic myeloid leukemia mice [26, 27]. Cancer cells frequently reprogram their metabolism to efficiently support cell proliferation and survival [28, 29]. However, whether and how these metabolic alterations contribute to insensitive HCC cells surviving sorafenib treatment remain largely unknown. Moreover, validated biomarkers for prediction of response to sorafenib have not been identified, although predictive values of a number of parameters have been studied [30, 31]. Thus, to improve the outcomes in HCC patients treated with sorafenib, it is critical to understand the molecular machinery that regulates the survival of insensitive HCC cells that escaped from sorafenib inhibition.

In this study, due to the heterogeneous sensitivity of tumor cells to sorafenib, we generated low-insensitive (LI), middle-insensitive (MI) and high-insensitive (HI) hepatocellular carcinoma cells surviving long-term exposure to various concentrations of sorafenib. We were very interested in understanding what changes have occurred in these surviving cells (LI, MI and HI cells). Therefore, we used tandem mass tag (TMT)-based quantitative proteomics to describe global intracellular protein changes in the LI, MI and HI cells. Our study unveils the proteomics landscape of the insensitive HCC cells, especially the core metabolic remodeling that supports their survival in high dosage of sorafenib. In particular, our findings uncovered the detailed mechanisms of sorafenib inhibition of mitochondrial holistic OXPHOS in human HCC cells.

## RESULTS

### Effects of sorafenib on HCC cell proliferation

**Figure 1:**
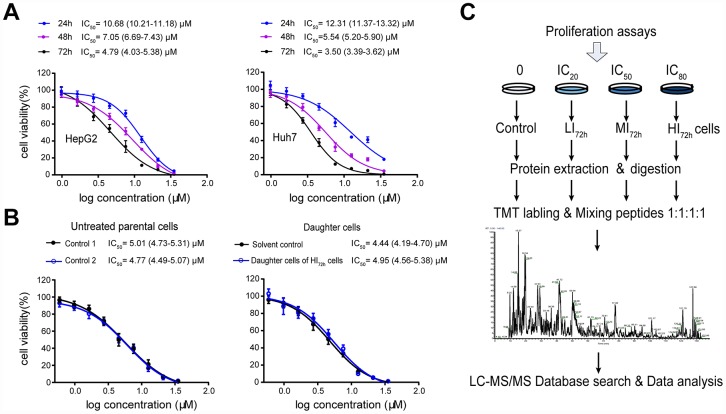
**High-insensitive HepG2 cells do not belong to the sorafenib-resistant HepG2 cells.** (**A**) Inhibitory effect of sorafenib treatment (24 h, 48 h and 72 h) on cell viability. The results represent as mean ± SD, n=6 per concentration. (**B**) Comparison of the IC_50_ values between untreated parental cells and the daughter cells of HI_72h_ HepG2 cells. Two groups of untreated cells were used as controls for DMSO treatment (solvent control) and IC_80_ of sorafenib treatment, respectively. The results represent as mean ± SD, n=5 per concentration. Dose response curves were analyzed via nonlinear regression followed by two-way ANOVA for comparisons between 2 curves, p> 0.05. (**C**) Workflow of TMT-based proteomic experiment. See Methods for experimental details.

Sorafenib inhibited the proliferation of HepG2 and Huh7 cells in a concentration- or time-dependent manner. The IC_50_ values following 24 h, 48 h and 72 h incubation in HepG2 cells were 10.68 (10.21-11.18) μM, 7.05 (6.69-7.43) μM and 4.79 (4.03-5.38) μM, respectively, and were 12.31 (11.37-13.32) μM, 5.54 (5.20-5.90) μM and 3.5 (3.39-3.62) μM, respectively, in Huh7 cells (Figure 1A). After 72 h incubation, the IC_20_, IC_50_ and IC_80_ values of sorafenib calculated by the probit regression analysis for HepG2 cells were 2.25 μM, 4.72 μM and 9.94 μM, respectively; the IC_20_, IC_50_ and IC_80_ values for Huh7 cells were 1.50 μM, 3.38 μM and 6.50 μM, respectively. HepG2 or Huh7 cells were cultured with 3 concentrations of sorafenib (IC_20_, IC_50_ and IC_80_) for 72 h incubation and the surviving cells were obtained as LI_72h_, MI_72h_ and HI_72h_ cells, respectively. These surviving cells were used in the subsequent proteomic studies or cell metabolism analysis.

To show qualities characteristic of these sorafenib insensitive cells, the IC_50_ of sorafenib was measured in the daughter cells of HI_72h_ HepG2 cells (Figure 1B), which were obtained by the 72 h incubation of HepG2 cells with sorafenib at IC_80_ (IC_80_ = 9.94 μM). When the daughter cells derived from HI_72h_ cells were re-exposed to sorafenib, the IC_50_ value was 4.95 (4.56-5.38) μM which was not significantly different from that (4.77 μM, 4.49-5.07 μM) in the untreated parental cells (Figure 1B).

### Proteomic analysis of LI_72h_, MI_72h_ and HI_72h_ HepG2 cells

### Proteomic data analysis by STEM software

**Figure 2:**
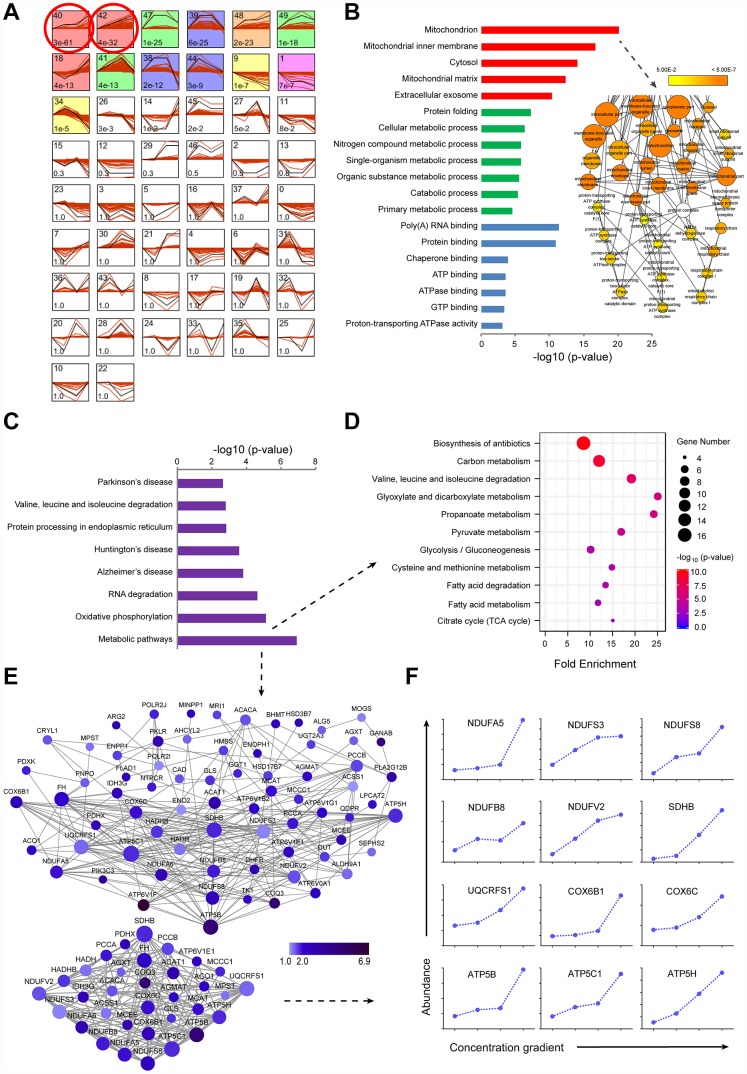
**TMT-based proteomic analysis of expression of identified proteins in LI_72h_, MI_72h_ and HI_72h_ HepG2 cells.** (**A**) Identification of clustered protein expression profiles by STEM. Model profiles (black) and the matched experimental protein expression profiles (red) are shown. Each box shows a profile identification number (upper left) and an unadjusted p value (lower left). Colored boxes represent model profiles assigned more proteins than expected by chance. (**B**) GO analysis of cellular components (red), biological process (green) and molecular function (blue) for 520 regulated proteins. 102 proteins involved in the term ‘mitochondrion’ were further analyzed by BiNGO plugin in Cytoscape. The node size corresponds to the number of proteins assigned to an individual term; p-value < 0.05 was defined as significant (yellow) and a darker color represents a lower p-value. See also supplementary Figure 2A and Table 2. (**C**) KEGG pathway analysis of 520 regulated proteins. (**D**, **E**) The proteins in the metabolic pathways were analyzed by the KEGG pathway (**D**, bubble chart) and the protein-protein interaction network (**E**). The extracted small network displays apparent interrelations between 33 mitochondrial proteins (lower panel, **E**). Each node is representative of a protein in the networks. The size of the nodes indicates the node degree that reflects the number of neighbors linked to a node. The color intensity corresponds to the fold change of proteins regulated in HI_72h_ cells compared to that in the control. (**F**) Quantified profiles of regulated proteins associated with mitochondrial oxidative phosphorylation in the control, LI_72h_, MI_72h_ and HI_72h_ HepG2 cells. See also Supplementary Table 3.

To gain a comprehensive overview of protein regulation by sorafenib, we used TMT-based quantitative proteomics to measure expression levels of global intracellular proteins in the LI_72h_, MI_72h_ and HI_72h_ HepG2 cells. General scheme of our current proteomic study was shown in Figure 1C. We have identified and quantified 4,100 proteins in HepG2 cells using the TMT-labeled proteomic method and a representative MS^2^ spectrum of the peptides is shown in Supplementary Figure 1A to indicate how the peptide sequences were identified by the Proteome Discoverer software (Version 2.1, Thermo Corporation, CA, USA). The maximal missed cleavage sites (K/R cleavage at C-terminus of the sequence), the TMT labeling efficiency and the distribution of special peptides for 4,100 proteins are shown in Supplementary Figure 1B-D. The proteomic data were normalized by the median ratio normalization (MRN) algorithm before the subsequent analysis and the effect of normalization is displayed in the IQR plots (Supplementary Figure 1E). STEM software was used to cluster the proteins according to the patterns of the identified proteins and to identify the host factors targeted by sorafenib in the LI_72h_, MI_72h_, and HI_72h_ cells and the control cells. Thirteen of 50 clusters were significantly enriched according to the STEM analysis (p<1×10^-5^, Figure 2A). There were 2 clusters with the highest enrichment (cluster^#40^ and cluster^#42^) with the p-values of p=3E^-61^ and p=4E^-32^, respectively; the quantification of protein expression in the 2 clusters involved 520 significantly regulated unique proteins and are positively related to the concentration of sorafenib (Supplementary Table 1).

### GO (Gene Ontology) analysis of 520 regulated proteins

The 520 proteins from cluster^#40^ and cluster^#42^ were classified by GO analysis according to cellular components (CC), biological processes (BP) and molecular functions (MF). The terms in CC, including mitochondrion, mitochondrial inner membrane, cytosol and mitochondrial matrix, were significantly enriched with the p-values less than 4.42 E^-12^; the p-value for the term ‘mitochondrion’ even reached 7.77E^-21^ (left panel, Figure 2B). Moreover, 102 proteins associated with the term ‘mitochondrion’ were analyzed by the Bingo plugin in Cytoscape and the results showed that these proteins were mainly distributed in the mitochondria (87%), mitochondrial respiratory chain (6.4%) and mitochondrial respiratory chain complex I (4.3%) (right panel of Figure 2B, Supplementary Figure 2A and Supplementary Table 2) indicating that a significant upregulation of the expression of mitochondrial proteins in LI_72h_, MI_72h_ and HI_72h_ cells is positively associated with sorafenib concentrations.

### KEGG (Kyoto Encyclopedia of Genes and Genomes) pathway analysis of 520 regulated proteins

Eight pathways, including metabolic pathways, oxidative phosphorylation, RNA degradation, etc., based on the KEGG pathway analysis were significantly enriched with p values less than 0.0028 (Figure 2C). A p-value of the term ‘metabolic pathways’ even reached 1.24E^-7^ and it included 80 proteins mainly participating in central carbon metabolism. These 80 proteins were reanalyzed using the KEGG pathway analysis and 11 pathways were significantly enriched with the p values less than 0.002, including carbon metabolism, pyruvate metabolism, glycolysis/gluconeogenesis, citrate cycle (TCA cycle), glyoxylate and dicarboxylate metabolism, propanoate metabolism, etc. (Figure 2D).

To gain a better understanding of the 80 proteins covered by the term ‘metabolic pathways’, a protein-protein interaction network was constructed based on the STRING database and visualized using Cytoscape; the average number of neighbors for each node was 7.099 (upper panel, Figure 2E) indicating close connections between these proteins. The interactions between 33 proteins associated with mitochondria were especially evident (lower panel, Figure 2E); 12 of the proteins in the small network were more closely related to mitochondrial respiratory chain, e.g., NDUFA5, NDUFB8, NDUFS3, NDUFS8 and NDUFV2 included in the subunits of CI and ATP5B, ATP5C1 and ATP5H included in the subunits of complex V (CV) (Figure 2F and Supplementary Table 3).

### Changes in cellular mitochondrial function and glycolysis in LI, MI and HI hepatocellular carcinoma cells

### OCR and ECAR measurements

**Figure 3:**
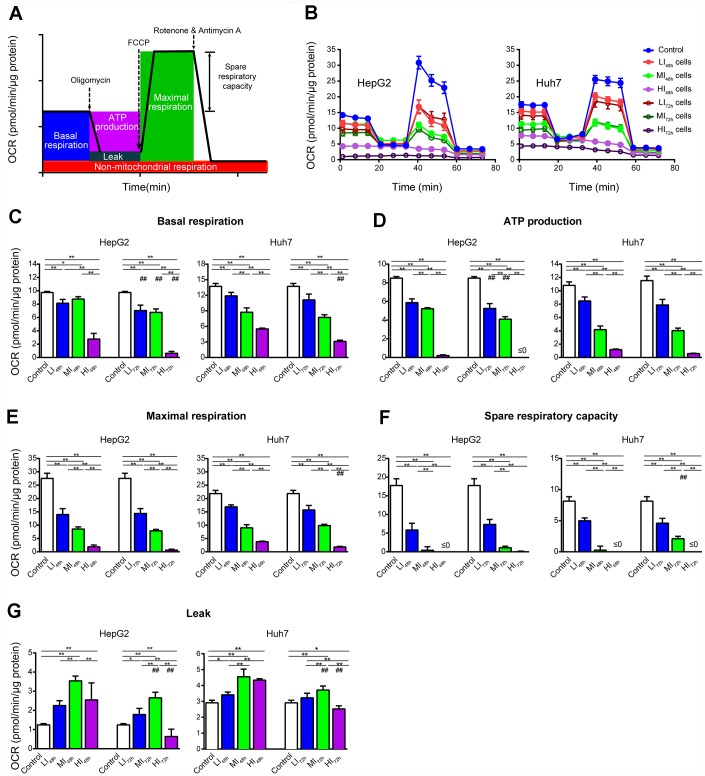
**Changes in OCR of LI, MI and HI hepatocellular carcinoma cells.** (**A**) Schematic of OCR analysis. (**B**) Representative OCR tracings of the control, LI, MI and HI cells at 48 h and 72 h. (**C**–**G**) The results of mitochondrial respiratory parameters for the control, LI, MI and HI cells at 48 h and 72 h. The results represent the mean ± SD, n=6 (except for HI_48h_ HepG2 cells with n=5); ^#^p < 0.05 and ^##^p < 0.01 compared to corresponding group at 48 h, *p < 0.05, **p < 0.01, and all statistical assessments were performed using one-way ANOVA followed by Dunnett′s test.

To understand the changes in cellular mitochondrial function and glycolysis in LI, MI and HI cells, OCR and ECAR were analyzed in these insensitive cells at 48 h and 72 h. Representative OCR tracings shown in Figure 3B demonstrate the effects of long-term treatment with sorafenib on HepG2 and Huh7 cells. Sorafenib significantly inhibited the basal respiration, ATP production, maximal respiration and spare respiratory capacity in a concentration-dependent manner in LI, MI and HI cells compared with those in the time-matched control cells. The maximal inhibition of these mitochondrial respiratory parameters in HepG2 cells were 93.5%, 100%, 97.6% and 99.8%, respectively (p < 0.01, Figure 3C–3F). Moreover, sorafenib exhibited stronger inhibitory effects on the basal respiration and ATP production in LI, MI and HI HepG2 cells at 72 h as compared to 48 h (p < 0.01). Huh7 showed a similar although less significant trend. Additionally, Leak respiration of MI and HI cells for both cell lines was all increased at 48 h compared to 72 h, especially in MI_48h_ HepG2 cells. Nearly 2-fold increase by sorafenib was found in MI_48h_ HepG2 cells compared with that in the time-matched control cells (p < 0.01, Figure 3G).

**Figure 4:**
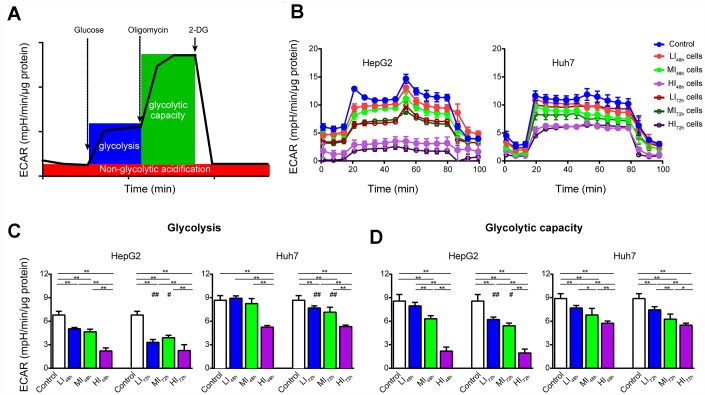
**Changes in ECAR of LI, MI and HI hepatocellular carcinoma cells.** (**A**) Schematic of ECAR analysis. (**B**) Representative ECAR tracings of the control, LI, MI and HI cells at 48 h and 72 h. (**C**, **D**) The results of glycolysis (**C**) and glycolytic capacity (**D**) in the control, LI, MI and HI cells at 48 h and 72 h. The results represent the mean ± SD, n=6; ^#^p < 0.05 and ^##^p < 0.01 compared to corresponding group at 48 h, *p < 0.05, **p < 0.01, and all statistical assessments were performed using one-way ANOVA followed by Dunnett′s test.

As shown by the representative ECAR tracings in Figure 4B, sorafenib significantly and concentration-dependently inhibited glycolysis and glycolytic capacity in LI, MI and HI HepG2 cells compared with those in the time-matched control cells with maximal inhibition by 67.6% and 77.4%, respectively (P < 0.01, Figure 4C, 4D). Additionally, the inhibition by sorafenib on LI and MI HepG2 cells was more marked at 72 h than at 48 h (p < 0.05). Similar inhibitory results were also observed in Huh7 cells. Interestingly, there was no significant difference in glycolysis and glycolytic capacity between HI_48h_ and HI_72h_ cells. (HepG2 and Huh7, p > 0.05).

### Cellular mitochondrial respiratory function measurements

**Figure 5:**
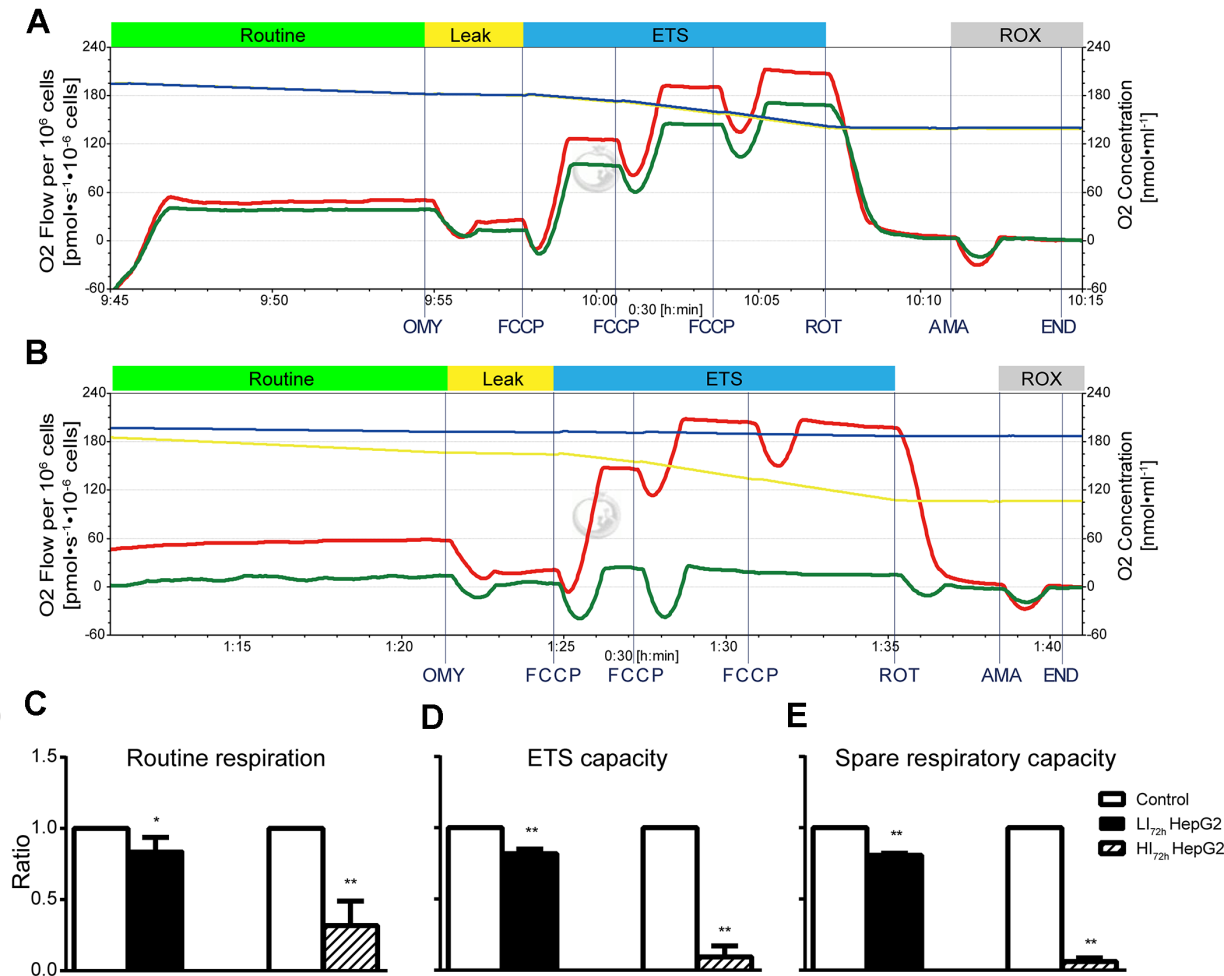
**Changes in cellular mitochondrial respiratory function of LI_72h_ and HI_72h_ HepG2 cells.** (**A**, **B**) Representative tracings of high-resolution respirometry in LI_72h_ (**A**) and HI_72h_ HepG2 cells (**B**) compared to the control cells, respectively. O2 flow per 10^6^ cells (left axis; green line for LI_72h_ or HI_72h_ cells and red line for control cells) and O2 concentration (right axis; blue line for LI_72h_ or HI_72h_ cells and yellow line for control cells) were recorded in real time. OMY, oligomycin; FCCP, carbonylcyanide-4-(trifluoromethoxy)-phenylhydrazon; ROT, rotenone; AMA, antimycin A. (**C**–**E**) The results of routine respiration (**C**), ETS capacity (**D**) and spare respiratory capacity (**E**) in LI_72h_ and HI_72h_ HepG2 cells. The results are presented as the mean ± SD; *p < 0.05, **p < 0.01 by unpaired, two-tailed Student’s t test. Experiments were repeated 3 times.

Changes in cellular mitochondrial respiratory function of LI_72h_ and HI_72h_ cells are shown in Figure 5A and Figure 5B (representative tracings). Consistent with OCR results (Figure 3), sorafenib significantly and concentration-dependently inhibited the routine respiration, ETS capacity, and spare respiratory capacity in LI_72h_ and HI_72h_ cells compared with that in control HepG2 cells and maximal inhibition was 68.3%, 90.7%, and 93.9%, respectively (p < 0.01, Figure 5C–5E).

### Effects of short-term treatment with sorafenib on mitochondrial respiratory function

**Figure 6:**
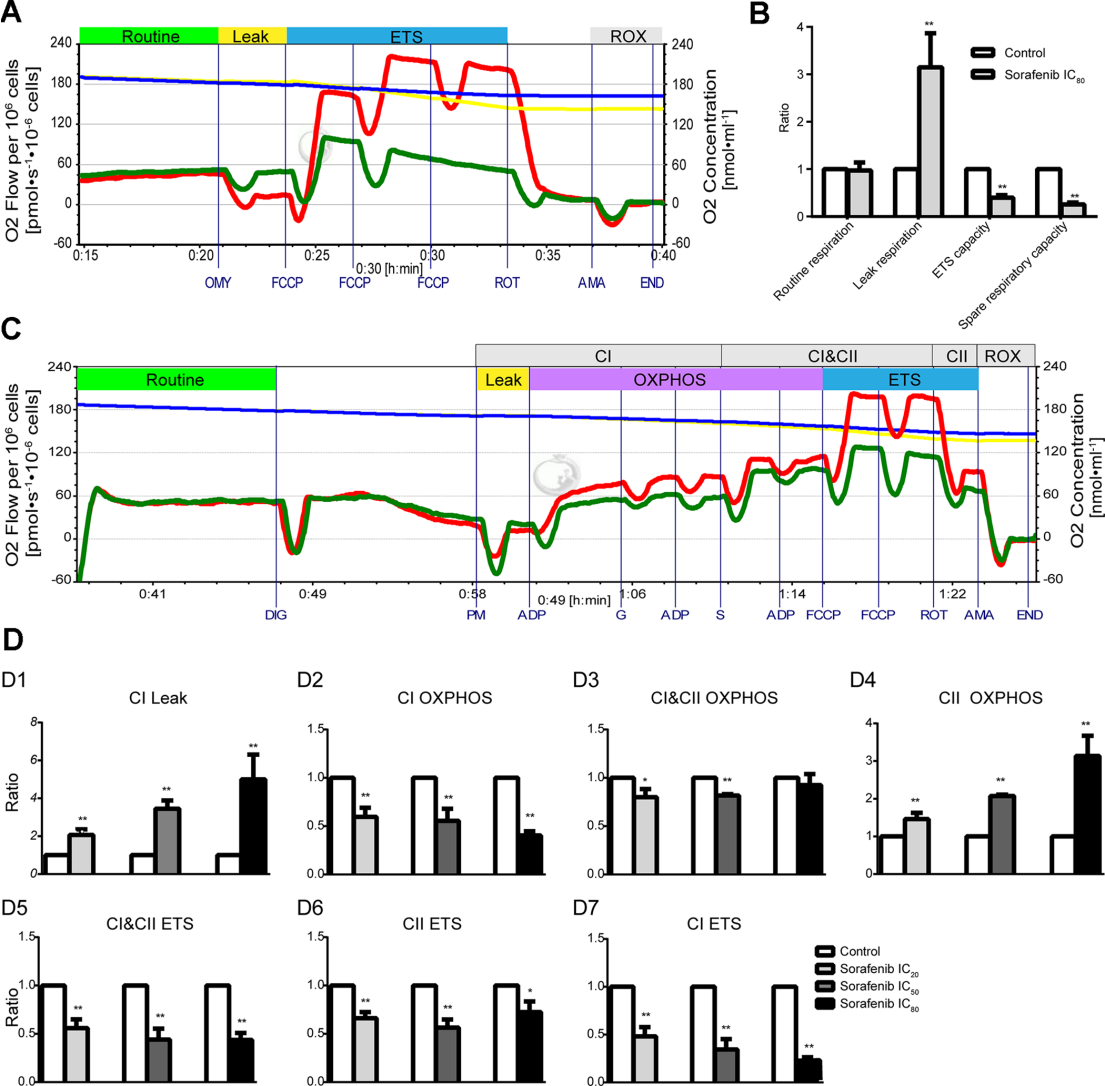
**Effects of short-term treatment with sorafenib on mitochondrial respiratory function in HepG2 cells.** (**A**, **B**) Representative tracings of high-resolution respirometry in intact HepG2 cells in the presence or absence of short-term treatment (15 min) with IC_80_ of sorafenib (**A**), and the statistical results of mitochondrial respiratory parameters (**B**). O2 flow per 10^6^ cells (green line for HepG2 cells in the presence of sorafenib and red line for control cells) and O2 concentration (blue line for HepG2 cells in the presence of sorafenib and yellow line for control cells) were recorded in real time. OMY, oligomycin; FCCP, carbonylcyanide-4-(trifluoromethoxy)-phenylhydrazon; ROT, rotenone; AMA, antimycin A. (**C**) Representative tracings of the effects of short-term treatment with IC_20_ of sorafenib on the OXPHOS system in permeabilized HepG2 cells. DIG, digitonin; PM, pyruvic acid + L-malic acid; G, glutamate; S, succinate; FCCP, carbonylcyanide-4-(trifluoromethoxy)-phenylhydrazon; ROT, rotenone; AMA, antimycin A; CI, Complex I; CII, Complex II; OXPHOS, oxidative phosphorylation; ETS, electron transfer system; ROX, residual oxygen consumption. (**D1–D7**) Summarized OXPHOS analysis. CI Leak, leak respiration of CI; CII OXPHOS = CI&CII OXPHOS − CI OXPHOS; CI ETS = CI&CII ETS − CII ETS. All data are presented as the mean ± SD; *p < 0.05, **p < 0.01 by unpaired, two-tailed Student’s t test. Experiments were repeated 3 times.

Effects of short-term treatment (15 min) with sorafenib on mitochondrial respiratory function in HepG2 cells are shown in the representative tracings in Figure 6A. Sorafenib at concentration corresponding to IC_80_ had no effect on routine respiration (p > 0.05); however, sorafenib significantly inhibited ETS capacity and spare respiratory capacity (p < 0.01) and significantly increased Leak respiration by 214.9% (p<0.01, Figure 6B). Moreover, the optimal concentration of FCCP to induce maximal respiration as the measure of ETS capacity in the presence of sorafenib at concentration corresponding to IC_80_ was reduced by approximately 2-fold compared with that in the time-matched control HepG2 cells.

Representative tracings of the effects of short-term treatment with sorafenib at concentration corresponding to IC_20_ on the OXPHOS system in permeabilized HepG2 cells are shown in Figure 6C. Sorafenib at three concentrations corresponding to IC_20_, IC_50_ and IC_80_ significantly enhanced Leak respiration of CI (CI Leak) in a concentration-dependent manner and the maximal enhancement was 399.8% (p < 0.01, Figure 6D1). Sorafenib significantly inhibited CI OXPHOS capacity and enhanced CII OXPHOS capacity and both effects were concentration-dependent (p < 0.01, Figure 6D2, 6D4); the maximal enhancement was 213.4%. Sorafenib significantly and concentration-dependently inhibited the CI ETS capacity and the maximal inhibition was 79.2% (p < 0.01, Figure 6D7). However, the significant inhibition of CI&CII ETS capacity and CII ETS capacity by short-term treatment with sorafenib was not concentration-dependent (p < 0.01, Figure 6D5, 6D6).

Supplementary Figure 3A shows the assay of cellular mitochondrial respiratory function using sorafenib instead of rotenone (specific inhibitor of CI) in HepG2 cells; dynamic changes in the oxygen fluxes induced by sorafenib were very similar to that induced by rotenone.

### Synergistic antiproliferative effects of long-term treatment with sorafenib in combination with 2-DG on Human HCC cells

**Figure 7:**
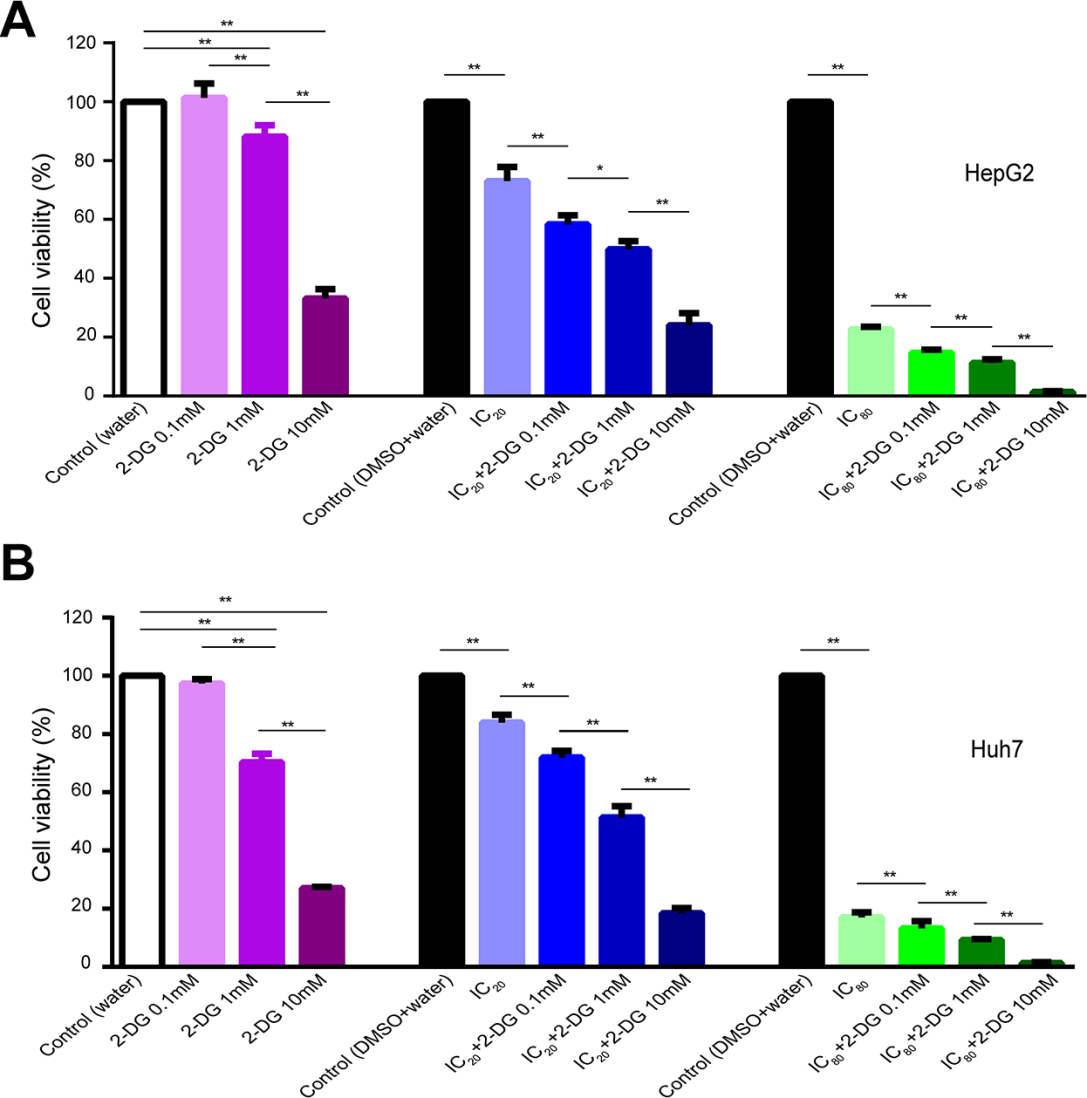
**Effect of 2-DG and sorafenib on human HCC cell proliferation.** (**A**, **B**) HepG2 cells (**A**) and Huh7 cells (**B**) were treated for 72 h with 2-DG alone, sorafenib alone, or in combination. Results are presented as the mean ± SD, n=4; *p < 0.05, **p < 0.01 using one-way ANOVA followed by Dunnett′s test.

ECAR results indicated longer time treatment of sorafenib did not further reduce glycolysis function of glycolysis and glycolytic capacity in HI cells (Figure 4); HI_72h_ cells might operate at their maximal glycolysis rate to energetically support compensatory. To obtain a synergistic antiproliferative effect in HCC cells, we tested the long-term treatment with sorafenib in combination with 2-DG. Single treatment of cells with 2-DG at 1 and 10 mM concentrations for 72 h significantly inhibited proliferation of HepG2 and Huh7 cells compared with that in the control (water) (Figure 7A, 7B). A long-term treatment with 10 mM 2-DG in combination with IC_20_ sorafenib or IC_80_ sorafenib inhibited HepG2 cell proliferation by 76.0% or 98.7%, respectively, compared to that in the corresponding control (water+DMSO) (p < 0.01), and inhibited the cell proliferation by 67.1% or 94.1%, respectively, compared with that in the case of the treatments with IC_20_ sorafenib or IC_80_ sorafenib (p < 0.01). Treatment with 0.1 mM 2-DG did not influence the proliferation of HepG2 and Huh7 cells. However, combination of 2-DG with IC_20_ sorafenib or IC_80_ sorafenib resulted in a substantial decrease in cell proliferation in both assays. HepG2 cells exhibited a decrease of 20.1% or 35.1%, respectively, compared with that in the case of the treatment with IC_20_ sorafenib or IC_80_ sorafenib (p < 0.01, Figure 7A).

## DISCUSSION

Insensitivity of HCC cells to sorafenib severely limits the outcome of cancer treatment. In our study the HepG2 cells surviving long-term-treatment with 3 concentrations of sorafenib, including LI_72h_, MI_72h_ and HI_72h_ HepG2 cells are insensitive to sorafenib. These surviving cells do not belong to the sorafenib-resistant HepG2 cells established by continuous exposure of HepG2 cells to increasing concentration of sorafenib [32]. Because the differentiated daughter cells of HI_72h_ cells had an IC_50_ value for sorafenib similar to that of the untreated HepG2 cells before sorafenib treatment. To explore the potential survival mechanism, we were the first to perform a large-scale proteomic analysis of the intracellular effects of various concentrations of sorafenib in cancer cells. Using a TMT-based quantitative approach, a total of 4,100 proteins were identified and quantified in the LI_72h_, MI_72h_ and HI_72h_ HepG2 cells; STEM analysis revealed two most enriched clusters, cluster^#40^ and cluster^#42^; in these clusters, 520 proteins were significantly and concentration-dependently upregulated by sorafenib in 3 types of cells. Analysis of significant quantitative changes in 520 proteins by the KEGG pathway indicated that 80 of the proteins are involved in the most significantly reliable term ‘metabolic pathways’ mostly related to central carbon metabolism, including carbon metabolism, pyruvate metabolism, glycolysis/gluconeogenesis, TCA cycle, etc. Specifically, protein-protein interaction analysis of these 80 proteins revealed that the majority of interactions between 33 proteins is associated with mitochondria and 12 of the proteins are involved in OXPHOS, including the ETC subunits of CI (NDUFA5, NDUFB8, NDUFS3, NDUFS8 and NDUFV2), CII (SDHB), CIII (UQCRFS1), complex IV (CIV) (COX6C and COX6B1), and CV (ATP5B, ATP5C1 and ATP5H). Consistently, GO enrichment analysis of 520 regulated proteins revealed that 102 mitochondrial proteins were significantly enriched (p=7.77E^-21^). Through a transcription factor (TF) prediction based on these 520 proteins, we identified several TFs such as NRF1 and YY1 (Supplementary Figure 2B, 2C), the canonical upstream regulators of mitochondrial genes, may take charge of the increased protein expression response to sorafenib treatment. Thus, these proteomic findings indicate that 102 mitochondrial proteins are incrementally upregulated in LI_72h_, MI_72h_ and HI_72h_ HepG2 cells; specifically, 12 of these proteins are closely related to mitochondrial OXPHOS and may play a role in the development of a survival mechanism to avoid antitumor activity of sorafenib.

Therefore, we speculated that HCC cells might survive the antitumor activity of high dose of sorafenib via remodeling of their mitochondrial respiratory function. Based on this hypothesis, we assessed the effect of long-term-treatment with sorafenib on cellular mitochondrial respiratory function of LI_,_ MI and HI cells at 48 h and 72 h. Despite the upregulation of numerous mitochondria proteins after sorafenib treatment, cellular mitochondrial respiratory function of HI hepatocellular carcinoma cells was significantly and very potently inhibited (Figures 3 and 5), and this finding was entirely unanticipated. It should be noted that even though the HI_72h_ cells were maintained in a state of the lowest basal respiration that was almost equal to the maximal respiration level, the cells preserved the ability to attach and proliferate after subculture. Mitochondrial respiratory function of the differentiated daughter cells of HI_72h_ cells was similar to that of the untreated HepG2 cells (data not shown). Since the levels of mitochondrial respiratory chain proteins in the LI_72h_, MI_72h_ and HI_72h_ cells were concentration-dependently increased according to our quantitative proteomics data, this contradiction raises a possibility that upregulation of mitochondrial respiratory chain proteins may be increased to compensate for severe repression of mitochondrial function. Eimre et al. [33] reported a similar phenomenon in WFS1-deficient mouse muscles with an increase in mitochondrial proteins that might compensate for a decrease in mitochondrial quality.

For a more detailed investigation of ETC, we used high-resolution respirometry to clarify possible mechanisms underlying mitochondrial respiratory inhibition or compensation. The concentrations of sorafenib used in the experiments with LI, MI and HI cells are clinically relevant [34]; hence, we investigated the effects of short-term treatment with sorafenib at the concentrations corresponding to IC_20_, IC_50_ or IC_80_ on HepG2 cells. Short-term treatment with sorafenib dramatically suppressed OXPHOS capacity and ETS capacity of CI in a concentration-dependent manner and maximal suppression was 59.7% and 79.2%, respectively (p < 0.01, Figure 6D2, 6D7). Bull et al. [35] used a method to determine oxidation of NADH to NAD^+^ that indirectly reflects the CI activity in human neuroblastoma cells and suggested that sorafenib impairs the CI activity. It should be specifically noted that in our study, sorafenib shows a rapid and direct inhibitory effect on CI function mimicking that of rotenone (Supplementary Figure 3A) most likely by inhibiting the electron transport from NADH dehydrogenase (CI) to coenzyme Q [36]. Moreover, we found that sorafenib serves as an uncoupling agent; this conclusion is supported by two types of evidence in the present study. First, consistent with the elevated proton leak in the Seahorse results, a short-term treatment with sorafenib at a concentration corresponding to IC_80_ induced a four-fold increase in Leak respiration of CI and a two-fold increase in Leak respiration compared with that in the time-matched control (Figure 6B, 6D1). Since Leak respiration is compensating mainly for proton leak after inhibition of ATP synthase. Second, the optimal concentration of FCCP titrated to obtain the maximum uncoupling capacity was reduced by two-fold in the HepG2 cells treated for 15 min with sorafenib at a concentration of IC_80_ (Figure 6A). It has been reported that certain mitochondrial uncoupling agents are able to reduce the coupling of OXPHOS by promoting proton leak across the mitochondrial inner membrane [37, 38] thus disrupting ATP production that depends on the coupling between CIV and CV [39] (Supplementary Figure 3B). In brief, sorafenib specifically targets mitochondrial CI and uncouples mitochondrial OXHPOS resulting in holistic inhibition of the OXHPOS function.

In contrast to the selective suppression of CI OXPHOS capacity, sorafenib treatment induced a compensatory increase in CII OXPHOS capacity in a concentration-dependent manner. This compensation coincided with the upregulation of a CII component (SDHB) in the proteomics analysis and may play an important role in the protection of cancer cells from the antitumor effect of sorafenib. Mitochondrial CII of the respiratory chain has a dual role linking the ETC and the TCA cycle [40] and CII inhibitors showed a promising anticancer activity through their proapoptotic and antiangiogenic potential; the compounds have been shown to synergize with anticancer agents inducing pleiotropic responses in cancer cells [41].

Considering the overwhelming inhibition of OXPHOS and ETS function by sorafenib, the majority of the compensatory expressed mitochondrial proteins expectedly failed to support the respiration and energy supply as shown in Figures 3 and 5 especially in the presence of the high dose sorafenib treatment. Glycolysis is another major bioenergetic pathway that participates in filling the gaps in ATP production. OCR and ECAR are known as an indicator of OXPHOS and an indicator of glycolysis, respectively [42], and the relative ratio of OCR/ECAR is often used as an indicator of potential metabolic switching. Usually, the OCR/ECAR ratio over 1 indicates a propensity to mitochondrial OXPHOS, while the ratio below 1 indicates a preference for glycolysis [43]. From the viewpoint of energy metabolism, sorafenib (IC_20_, IC_50_ and IC_80_) significantly and concentration-dependently inhibited glycolytic metabolism in LI_72h_, MI_72h_ and HI_72h_ cells confirming that sorafenib is an effective repressor of global energy metabolism in HCC cells (Figure 4). Nevertheless, HI_72h_ cells displayed a significant decrease in basal OCR compared with the values in HI_48h_ cells (77.2% and 43.8% decrease for HepG2 and Huh7 cells, respectively), but remained equal ECAR value. We also observed that HI_72h_ cells exhibited reduced basal OCR/ECAR ratios compared with HI_48h_ cells (Supplementary Figure 4A, 4B), suggesting higher reliance of the HI cells on glycolysis to maintain their energy requirements. Consistently, the KEGG pathway analysis revealed that certain proteins involved in the glycolytic pathway were concentration-dependently upregulated. A previous study reported that sorafenib at a concentration less than 10 μM after 24 h treatment inhibited OXPHOS but did not inhibit glycolysis in HepG2 cells [44] corroborating the observation that mitochondrial function is the priority target of sorafenib. Therefore, it was reasonable to assume that HI_72h_ cells that underwent a metabolic switch from mitochondrial OXPHOS to glycolysis avoid the inhibitory effect of high dose of sorafenib and that metabolic reprogramming is able to provide basic ATP to support the tumor cell survival. Thus, to confirm whether the metabolic reprogramming can support the survival of insensitive cells, the effects of a combination of 2-DG with long-term treatment with sorafenib were investigated. As expected, 2-DG sensitizes HCC cells to cell death induced by sorafenib and synergizes with sorafenib. Even explained the mechanisms underlying the recent observation that the combination of sorafenib and HK2 silencing increased HCC cell death, and synergistically inhibited tumor growth [45].

In summary, we are the first to demonstrate that sorafenib, an FDA-approved drug for HCC, remarkably suppresses the overall mitochondrial OXPHOS of human HCC cells by two different mechanisms: directly targeting the CI-linked electron transport and OXPHOS capacity and blocking ATP production coupled to respiration as a potential uncoupler. However, there was always a certain number of HCC cells insensitive to the long-term treatment with sorafenib even at the highest concentration of the drug, and these cells survived via metabolic reprogramming including extensive compensatory regulation of the bioenergetic pathway proteins. First, major reprogramming was identified as a compensatory increase in massive mitochondrial proteins, especially in the ETS components, including the maintenance of the expression levels of the CI subunits and an increase in abundance and activity of CII that may bypass the inhibition of CI electron transport by sorafenib. Another mechanism included metabolic switch from OXPHOS to glycolysis when the former was restrained by high dose of sorafenib, thus maintained energy supply to support the survival of cancer cells. The paradoxical sensitivity and stable dose-dependent compensation of the ETS components under sorafenib treatment, especially the upregulation of complex I and II subunits, may serve as a reliable indicator that predicts the drug response and prognosis of sorafenib treatment.

## MATERIALS AND METHODS

### Reagents

Fetal bovine serum (FBS), minimum essential medium (MEM), Dulbecco’s modified essential media (DMEM), sodium pyruvate, and L-glutamine were from Gibco (California, USA); sorafenib was from ApexBio (Houston, USA); CCK-8 was from Dojindo Corporation (Shanghai, China); dithiothreitol (DTT) was from BBI Life Sciences Corporation (Shanghai, China); EGTA was from Takara (Ohtsu, Japan); Lys-C and trypsin were from Promega Corporation (Shanghai, China). Iodoacetamide (IAM), triethylammonium bicarbonate (TEAB), oligomycin, carbonylcyanide-4-(trifluoromethoxy)-phenylhydrazon (FCCP), rotenone, antimycin A, 2-deoxy-D-glucose (2-DG), glutamate, pyruvic acid, L-malic acid, succinate, adenosine diphosphate (ADP), MgCl_2_·6H_2_O, lactobionic acid, KH_2_PO_4_, D-sucrose, taurine, HEPES and BSA were obtained from Sigma-Aldrich Corporation (Shanghai, China).

### Cell culture

The human HCC cell lines HepG2 and Huh7 were purchased from Cell Bank of Chinese Academy of Sciences (Shanghai, China). HepG2 cells were cultured in MEM containing 5.55 mM D-glucose supplemented with 10% FBS, 2 mM glutamine, 100 units·ml^-1^ penicillin and 100 μg·ml^-1^ streptomycin. Huh7 cells were cultured in DMEM containing 10 mM D-glucose supplemented with 10% FBS, 100 units·ml^-1^ penicillin and 100 μg·ml^-1^ streptomycin. Cultures were grown at 37 °C in a 5% CO_2_ environment and were passaged every 3 days thus increasing the passage number. Cells at passages 3-6 were used in the present study.

### Proliferation assays

### Effects of sorafenib on human HCC cell proliferation

Cell viability was determined using the Cell Counting Kit-8 (CCK-8) assay. Cells were seeded in 96-well plates at a density of approximately 5 × 10^3^ cells/well and were allowed to firmly attach to the surface; then, the medium was changed to the sorafenib-containing medium. Sorafenib was dissolved in DMSO at a concentration of 10 mM and diluted with the culture medium to the final concentrations from 0.6 to 35 μM in the sorafenib-containing media. Experiments were performed in six replicate wells for each concentration. After 72 h incubation with sorafenib, the sorafenib-containing medium was replaced with fresh medium containing CCK-8 solution (10:1, *v/v*) and the incubation was continued for 2 h in the dark. Absorbance was measured at 450 nm using a microplate reader (Fluostar Omega, BMG Labtech Ltd.). The dose-response curves were fit to a nonlinear regression and the half-maximal inhibitory concentration (IC_50_) for sorafenib was calculated using the GraphPad Prism 6.00 software (GraphPad Software Inc, USA). Then IC_20_, IC_50_ and IC_80_ values of sorafenib that inhibited cell growth by 20%, 50% and 80%, respectively, were calculated by the SPSS software (version 21.0; SPSS, USA). The concentrations of sorafenib corresponding to IC_20_, IC_50_ and IC_80_ were used in the subsequent proteomic studies and cell metabolism analysis.

### Viability of the HepG2 cells that survived long-term incubation with IC_80_ of sorafenib

HepG2 cells were seeded in several 75-cm^2^ culture flasks at a density of approximately 6×10^6^ cells per flask and divided into 4 groups after cells were attached. A group of cells was incubated with IC_80_ of sorafenib for 72 h and then, the surviving cells were collected and thoroughly washed with phosphate buffered saline (PBS). The surviving cells (HI_72h_ cells) were continually cultured in a drug-free medium for another 48 h recovery. In the second group, HepG2 cells were treated in the same manner except that sorafenib was replaced with an equivalent concentration of DMSO (solvent control). The other two groups without any treatment served as a pretreatment control for sorafenib and DMSO treatment groups, respectively. CCK-8 assay was used to compare the changes in the IC_50_ values of sorafenib between the subculture of the surviving cells and the untreated parental cells.

### Proteomics study in HepG2 cells surviving long-term incubation with IC_20_, IC_50_ and IC_80_ of sorafenib

### Protein sample preparation

As described in Figure 1C, HepG2 cells were cultured with IC_20_, IC_50_ and IC_80_ of sorafenib for 72 h. The HepG2 cells that survived the long-term incubation with 3 concentrations of sorafenib were carefully collected and are referred as LI_72h_, MI_72h_, and HI_72h_ HepG2 cells, respectively. Intact cell samples from 3 types of HepG2 cells including the solvent control group were washed twice in PBS and harvested with a cell scraper. The samples were then lysed in 8 M urea (8 M urea, 50 mM Tris-HCl, pH 8.0) with a brief sonication to promote protein solubilization. The protein supernatants were collected and protein concentration in each sample was determined by the bicinchoninic acid (BCA) assay.

In each sample of the 4 groups, 30 μg of the extracted protein was reduced by incubation with 20 mM DTT for 1 h and alkylated by incubation with 50 mM IAM for 45 min at 37 °C in the dark. Then, the proteins were precipitated overnight with ice-cold acetone and pelleted by centrifugation at 8 ×10^3^ g at 4 °C for 10 min. For the digestion step, the protein pellets were redissolved in 100 μl of 50 mM TEAB and digested with Lys-C enzyme (enzyme:substrate = 1:150, *w/w*) at 37 °C for 3 h; digestion was continued by adding trypsin (enzyme:substrate = 1:100, *w/w*) at 37 °C overnight. Finally, the digestion was terminated by adding 10 μl of 10% formic acid (FA) to 1% final concentration. The digested peptides were dried after centrifugation at 2 ×10^4^ g at 4 °C for 10 min and stored at -80 °C before use.

### Tandem mass tag (TMT) labeling and fractionation

For TMT labeling, the peptide samples of the 4 groups were separately labeled using 4 labeling reagents of the 6-plex label sets (Thermo Scientific, USA). Each sample containing 30 μg protein was resuspended in 50 μl TEAB buffer containing 60% acetonitrile (ACN) and mixed with 20 μl of the corresponding TMT reagent for 2 h at 26 °C. The labeling reactions were then quenched by adding 5% hydroxylamine for an additional 30 min incubation. Four labeled peptide samples were mixed in equal amounts and dried in a centrifugal evaporator.

The mixed TMT-labeled peptides were resuspended in 110 μl of mobile phase A (20 mM ammonium formate buffer, 3% ACN, pH 10.0) and separated using a 1.7 μm × 2.1 mm × 100 mm BEH C18 column (Waters, USA) in a Dionex Ultimate 3000 RSLC system with a 60 min gradient starting from 4% mobile phase B (ACN) to 64% B at a flow rate of 0.25 ml·min^-1^. Eluted fractions were pooled into 10 peptide samples over the gradient at 1 min intervals and were dried to completion. The peptide samples were reconstituted in 0.1% formic acid for the subsequent LC-MS/MS analysis.

### LC-MS/MS analysis

The TMT-labeled peptides were analyzed by a Thermo Easy-nLC 1000 HPLC system coupled with an Orbitrap Fusion mass spectrometer using a NanoFlex source (Thermo Scientific, USA). Chromatographic separation and the MS method were described previously [46]. Briefly, the peptides were trapped with a PepMap100 C18 trapping column (75 μm × 2 cm, Thermo Scientific, USA) and eluted from an Acclaim PepMap C18 RSLC column (1.7 μm, 75 μm × 15 cm, Thermo Scientific, USA) with buffer A (0.1% formic acid) and buffer B (ACN, 0.1% formic acid) gradient during 140 min; the gradient was delivered at a flow rate of 300 nl·min^-1^. The gradient was as follows: 5% B from 0 to 3 min, 5–7% B from 3 to 5 min, 7–25% B from 5 to 105 min, 25–60% B from 105 to 125 min, 60–95% B from 125 to 126 min, and 95% B from 126 to 140 min.

An Orbitrap Fusion mass spectrometer was operated in the data-dependent mode for the MS^2^ data acquisition. For the MS^1^ method, a full scan was acquired in the range of 350-1550 m/z at the resolution of 12×10^4^. Ion filtering for MS^2^ events was isolated by a quadrupole with a transmission window of 1.6 m/z. High energy collisional dissociation (HCD) fragmentation was performed with 37% normalized collision energy followed by analysis of the fragment ions in the Orbitrap with a resolution of 3×10^4^ at m/z 200. The duty cycle was fixed at 3 s. The automatic gain control (AGC) settings were 2 × e^5^ and 5 × e^4^ ions and maximal ion injection times of 50 and 100 ms were set for full and MS^2^ scans, respectively.

### Protein identification and quantification and bioinformatic analysis

Raw data were analyzed with the Proteome Discoverer 2.1 software (Thermo Scientific, USA). SEQUEST HT search engine with raw instrument files searched against the Homo species proteome database (updated on July 2018; 52,704 proteins) with the addition of reversed sequence decoy strategy to calculate the false discovery rates. Default settings were used with an exception of allowing 2 missed cleavages; a parent ion tolerance of 10 ppm and a fragment mass tolerance of 0.02 Da were set; a false discovery rate (FDR) cutoff value of 1% was applied at the peptide and protein levels. Carbamidomethyl (+57.021 Da) was selected as a fixed modification and the TMT reagents (+229.163 Da) coupled to lysines, oxidation of methionine residues (+15.9949 Da) and N-terminal acetylation (+42.011 Da) were selected as variable modifications.

The Short Time-series Expression Miner (STEM) software was used to cluster and visualize the gene expression profiles. Subsequently, functional enrichment analysis of the identified proteins, including KEGG (Kyoto Encyclopedia of Genes and Genomes) pathway analysis and GO (Gene Ontology) analysis for biological processes (BP), molecular functions (MF) and cellular components (CC), was conducted using the DAVID online platform (https://david.ncifcrf.gov). To detect functional coupling and to visualize protein interactions between the regulated proteins influenced by 3 concentrations of sorafenib treatment, we used the Cytoscape software / Biological Network Gene Ontology (BiNGO) plugin in Cytoscape based on the STRING database. In addition, transcription factors (TFs) targeting differentially expressed genes were predicted using the online tools Pscan [47] with the Jaspar database and Cscan [48] with the HepG2 ChIP-seq experiment data. The analysis focused on the genes upstream transcription start site (TSS) between nucleotides −450 and +50.

### Metabolic analysis of HCC cells surviving long-term incubation with IC_20_, IC_50_ and IC_80_ of sorafenib

### Extracellular flux analysis

For metabolic analysis, oxygen consumption rate (OCR) and extracellular acidification rate (ECAR) in HepG2 and Huh7 cells were measured using a Seahorse XFe-96 extracellular flux analyzer (Seahorse Bioscience, Agilent, USA). HepG2 and Huh7 cells were cultured with sorafenib at the concentrations corresponding to IC_20_, IC_50_ and IC_80_. The cells surviving long-term incubation (at 48 h or 72 h) with 3 concentrations of sorafenib are referred as LI_48h_, MI_48h_, HI_48h_, LI_72h_, MI_72h_ and HI_72h_ cells, respectively. In these groups and the solvent control group, fresh cells were seeded in Seahorse XFe-96 plates at an optimized density to achieve similar confluence after sorafenib treatment. A density of 8×10^3^ cells/well for HepG2 or 3×10^3^ cells/well for Huh7 was used for solvent control, LI and MI cell groups, whereas 4×10^4^ cells/well for HepG2 or 1.8×10^4^ cells/well for Huh7 was used in the case of HI cell groups. The cells surviving long-term incubation with sorafenib were washed with assay medium (Seahorse XF Base Medium supplemented with 5.5 mM glucose, 2 mM L-glutamine and 1 mM sodium pyruvate) and incubated at 37 °C without CO_2_ for 1 h. For a mito-stress test, sequential injections of 2 μM oligomycin, 0.75 μM FCCP for HepG2 cells or 0.4 μM FCCP for Huh7 cells, 1 μM antimycin A and 1 μM rotenone were performed during real-time OCR recording. For a glycolysis stress test, ECAR was performed in Seahorse XF base medium supplemented with 2 mM L-glutamine as the baseline conditions and sequential injections of 10 mM glucose, 2 μM oligomycin and 100 mM 2-DG were performed. Immediately after each run, the cells plated in XFe-96 plates were lysed in RIPA buffer and the total protein concentration was determined using the BCA assay for normalization. The experiments were performed in six replicate wells for each group.

### Cellular mitochondrial respiratory function measured by high-resolution respirometry

After a long-term incubation, the LI_72h_ and HI_72h_ HepG2 cells were carefully washed twice in PBS and harvested via trypsin digestion; the cells were then resuspended in MEM culture medium at a cell density of 5×10^5^ cells·ml^-1^ in 2-ml chambers stirred at a speed of 750 rpm. The cellular mitochondrial respiratory rate was measured at 37 °C with a high-resolution mitochondrial respirometer (Oxygraph-2k, Oroboros Instruments, Innsbruck, Austria) [49]; real-time data acquisition of O_2_ concentration (nmol·ml^-1^) and O_2_ flow per 10^6^ cells (pmol·s^-1^·10^-6^ cells) were performed using the DataLab software (Oroboros Instruments, Innsbruck, Austria). Routine respiration was measured when the respiration was stabilized. After the addition of 2.5 μM oligomycin, mitochondrial respiration declined to the leak-compensating state. Stepwise titrations with FCCP at 5 μM increments to an optimal concentration were performed to estimate the electron transfer system (ETS) capacity. Residual oxygen consumption (ROX) was measured after addition of 0.5 μM rotenone and 2.5 μM antimycin A. Spare respiratory capacity was calculated by subtracting routine respiration from the ETS capacity. Each experiment was repeated 3 times.

### Effects of short-term treatment with sorafenib on mitochondrial respiratory function in HepG2 cells

Cultured HepG2 cells without drug treatment were used to observe the effects of short-term treatment (15 min) with sorafenib on cellular mitochondrial energy metabolism using an Oxygraph-2k high-resolution respirometer. Two equal samples of HepG2 cell suspension were separately transferred to two sample chambers; one chamber was used for sorafenib treatment at a concentration of sorafenib corresponding to IC_80_ and the other chamber was used as a solvent control. After short-term treatment with sorafenib, 2.5 μM oligomycin, FCCP titrations in 5 μM steps, 0.5 μM rotenone and 2.5 μM antimycin A were sequentially added to the sample chambers; the related parameters (routine respiration, Leak, ETS capacity, and spare respiratory capacity) were recorded.

Cultured HepG2 cells without drug treatment were used to observe selective targets of short-term treatment with sorafenib at 3 concentrations corresponding to IC_20_, IC_50_ and IC_80_ using a specifically designed substrate-uncoupler-inhibitor titration (SUIT) protocol for detection of mitochondrial respiratory chain in permeabilized cells using the same instrument [50]. Briefly, the cells harvested via trypsin digestion were rinsed twice and diluted with respiration medium MIR05 (containing 0.5 mM EGTA, 3 mM MgCl_2_·6H_2_O, 60 mM lactobionic acid, 20 mM taurine, 10 mM KH_2_PO_4_, 20 mM HEPES, 110 mM D-sucrose and 1 g/L BSA; pH 7.0) to 5×10^5^ cells∙ml^-1^. After the cells were incubated with sorafenib at indicated concentration for 10 min in the sample chambers, an optimal concentration of digitonin (5 μg·10^-6^ cells) was added to permeabilize the cell membrane for 5 min. CI-linked Leak (CI Leak) respiration was measured after adding 5 mM pyruvic acid and 2 mM L-malic acid in the absence of ADP. A saturating concentration of 5 mM ADP was added to activate CI OXPHOS and the CI OXPHOS capacity was determined after the addition of 10 mM glutamate and 5 mM ADP. Subsequent addition of 10 mM succinate allowed to measure the convergent OXPHOS capacity through both CI and complex II (CII) (CI&CII OXPHOS capacity). CI&CII ETS capacity was evaluated by uncoupling of OXPHOS by stepwise titration with FCCP in 2.5 μM steps to an optimum concentration. CII ETS capacity was measured by adding 0.5 μM rotenone for inhibition of CI. ROX was obtained by inhibition of complex III (CIII) with 2.5 μM antimycin A. All experiments were repeated 3 times.

### Synergistic antiproliferative effects of long-term treatment with sorafenib in combination with 2-DG on human HCC cells

This part of the study was designed to evaluate the synergistic antiproliferative effects of sorafenib in combination with 2-DG on cultured HepG2 and Huh7 cells in 13 experimental groups including 0.1 mM 2-DG, 1 mM 2-DG, 10 mM 2-DG, sorafenib IC_20_, sorafenib IC_80_, distilled water, water+DMSO, sorafenib IC_20_ in combination with 0.1 mM 2-DG, sorafenib IC_20_ in combination with 1 mM 2-DG, sorafenib IC_20_ in combination with 10 mM 2-DG, sorafenib IC_80_ in combination with 0.1 mM 2-DG, sorafenib IC_80_ in combination with 1 mM 2-DG and sorafenib IC_80_ in combination with 10 mM 2-DG. Cultured cells without drug treatment were seeded in a 96-well plate (5 ×10^3^ cells/well) and allowed to firmly attach to the surface; then, the culture medium was replaced with the indicated additions corresponding to 13 experimental groups described above; the culture was continued for 72 h. CCK-8 assay was performed to detect cell viability. Testing of each group was carried out in quadruplicate and repeated three times.

### Data and statistical analysis

Data are presented as the mean ± SD. Two-way ANOVA was used to compare the differences between the two sets of the dose-response curves using the GraphPad Prism 6.00 software. The identified proteins were visualized and analyzed using Cytoscape and R language (R 3.5.2). Unpaired, two-tailed Student’s t test was used to examine the differences between two groups and one-way ANOVA followed by Dunnett′s test was used to analyze more than two groups in the case of metabolic assays and synergistic effects of sorafenib in combination with 2-DG. Data were analyzed using the GraphPad Prism 6.00 software. A P value less than 0.05 was considered statistically significant.

## Supplementary Material

Supplementary Figures

Supplementary Table 1

Supplementary Table 2

Supplementary Table 3
