# Supplementary material for: Mitochondrial metabolic study guided by proteomics analysis in hepatocellular carcinoma cells surviving long-term incubation with the highest dose of sorafenib
Source: Aging (Albany NY). 2019 Dec 26;11(24):12452–75. doi: 10.18632/aging.102582 (PMC6949094; doi:10.18632/aging.102582)
Supplement: Supplementary Figures [file aging-11-102582-s004..pdf]

SUPPLEMENTARY FIGURES

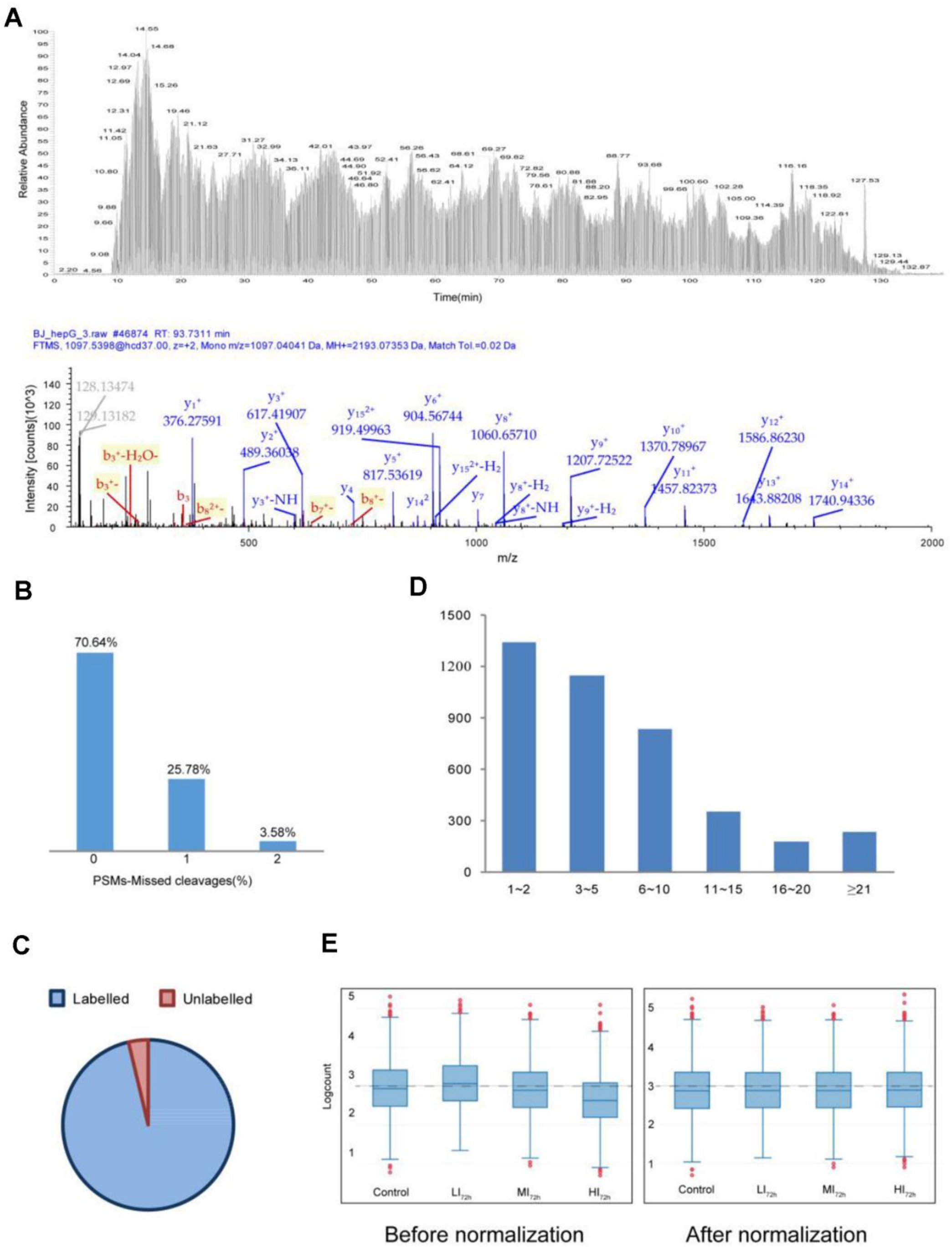

Supplementary Figure 1. Proteins identified and quantified using the nLC-MS/MS method. (**A**) A representative MS^1 and MS^2 spectrum detected by an Orbitrap fusion mass spectrometer. (**B**) The percentage of the peptide-spectra matches (PSMs) with zero to two missed cleavages employing consecutive proteolytic digestion with Lys C and trypsin. (**C**) Labeling efficiency of peptides by TMT reagents. (**D**) The distribution of special peptides for 4,100 proteins. (**E**) Data normalization effect by the median ratio normalization (MRN) algorithm.

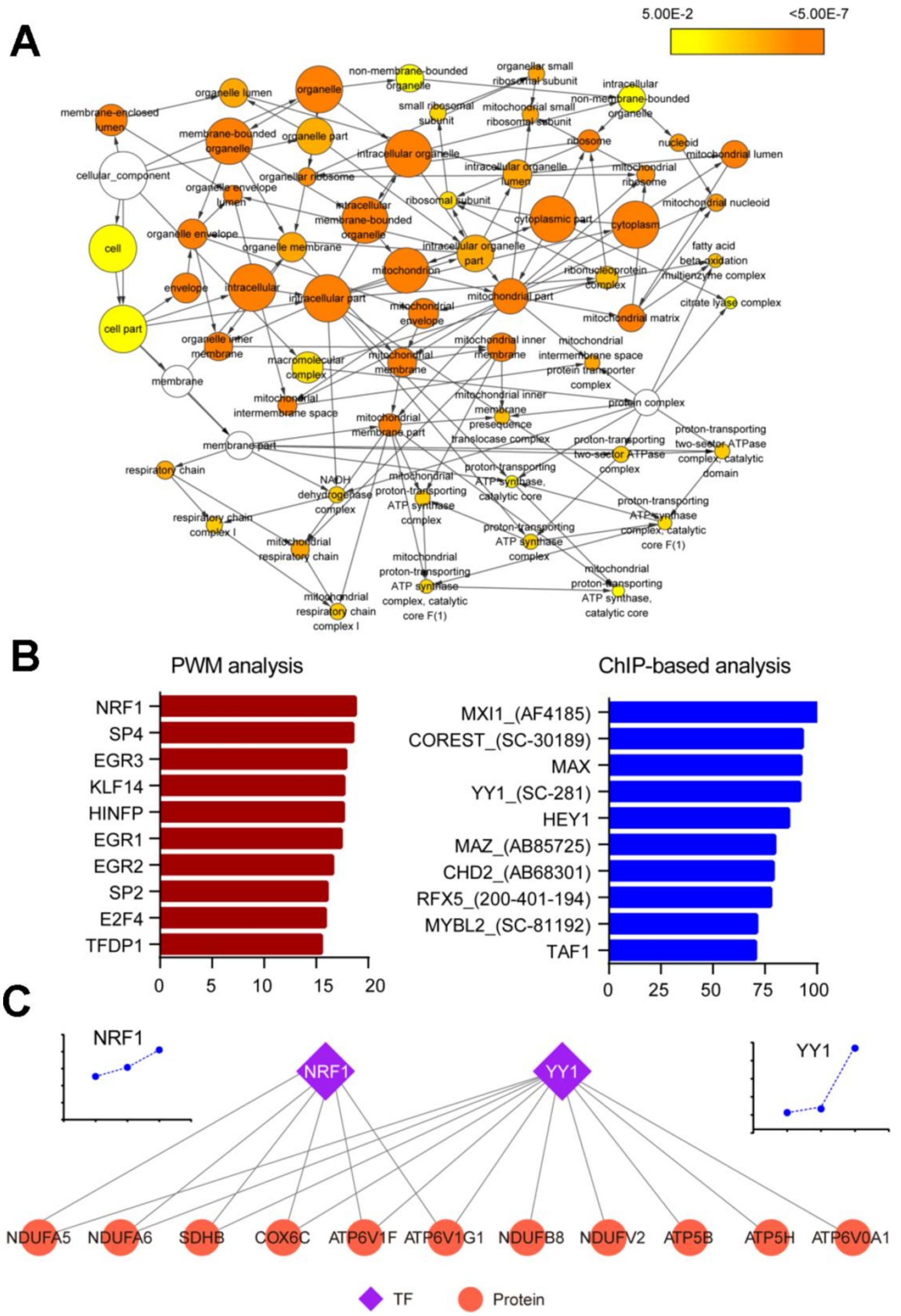

Supplementary Figure 2. GO analysis for 102 proteins involved in 'mitochondrion' term and upstream transcription factors (TFs) prediction of 520 regulated proteins. (**A**) GO analysis for 102 proteins involved in 'mitochondrion' term using BiNGO plugin in Cytoscape related to Figure 2B. The node size corresponds to the number of proteins assigned to an individual term; p-value < 0.05 was defined as significant (yellow); a darker color represents a lower p-value. (**B**) Top-ranked upstream TFs prediction from the 520 proteins in cluster$^{\#40}$ and cluster$^{\#42}$ using promoter weighted matrix (PWM) and ChIP-based methods. (**C**) The TF-target regulatory network of mitochondria related TFs and the upregulated proteins in the KEGG term 'oxidative phosphorylation'. TFs and targets are represented by diamonds and circles, respectively. Proteomic quantitation of NRF1 and YY1 are shown.

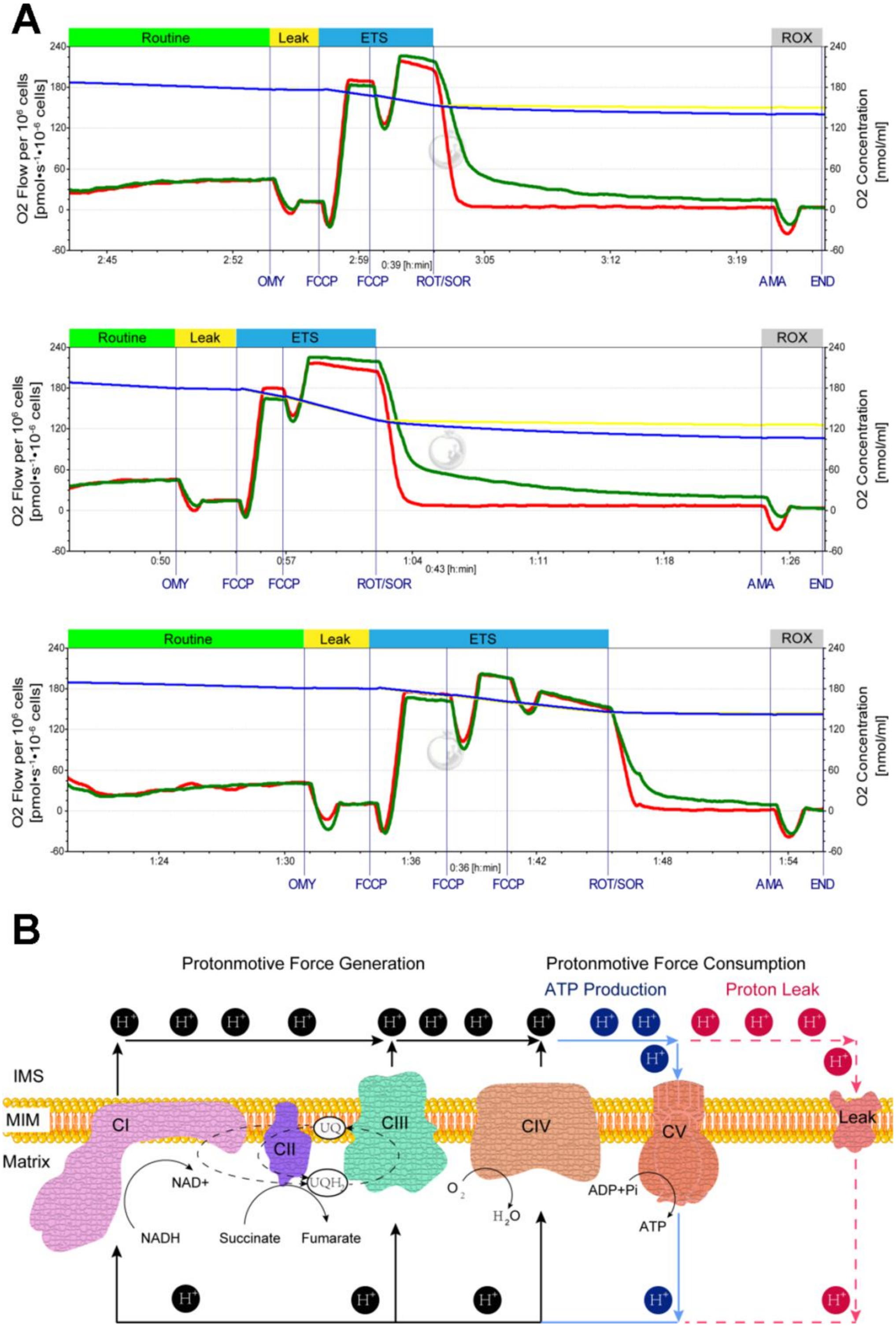

Supplementary Figure 3. Cellular mitochondrial respiratory experiment performed by replacing rotenone with sorafenib. (**A**) Representative tracings of high-resolution respirometry in HepG2 cells using sorafenib instead of rotenone. Red line, O2 flow in response to the application of rotenone, an inhibitor of complex I that inhibits electron transport from complex I to coenzyme Q; green line, O2 flow in response to the application of sorafenib. The dynamic changes in the oxygen fluxes induced by sorafenib were very similar to that induced by rotenone. OMY, oligomycin; FCCP, carbonylcyanide-4-(trifluoromethoxy)-phenylhydrazon; ROT, rotenone; SOR, sorafenib; AMA, antimycin A. (**B**) Schematic representation of the electron transfer system (ETS) coupled to the phosphorylation system and proton generation and consumption.

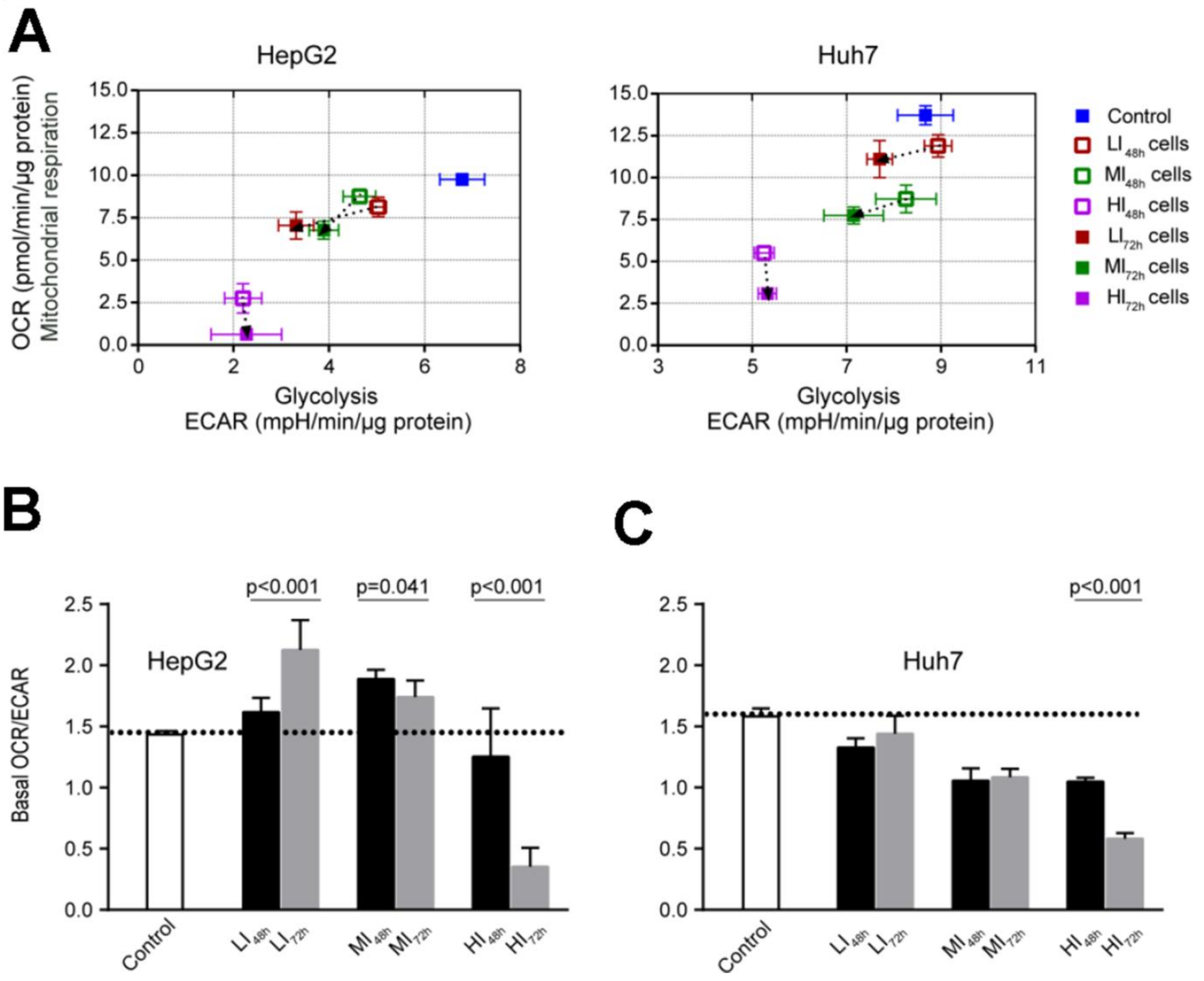

Supplementary Figure 4. Cellular energy phenotype analysis of LI, MI and HI hepatocellular carcinoma cells. (**A**) Cellular energy phenotype profiles of the control, LI, MI and HI cells. Basal OCR and ECAR of the control, LI, MI, HI cells at 48 h and 72 h are compared in a 2D plot to illustrate relative utilization of two energy production pathways (mitochondrial respiration and glycolysis). (**B**) Basal OCR/ECAR. The results are presented as the mean ± SD, n=6 (except for HI_{48h} HepG2 cells with n=5). Statistical assessments were performed using unpaired, two-tailed Student's t test.
