# Supplementary material for: Mitochondrial metabolic study guided by proteomics analysis in hepatocellular carcinoma cells surviving long-term incubation with the highest dose of sorafenib
Source: Aging (Albany NY). 2019 Dec 26;11(24):12452–75. doi: 10.18632/aging.102582 (PMC6949094; doi:10.18632/aging.102582)
Supplement: Supplementary Table 1 [file aging-11-102582-s002..docx]

**Supplementary Table 1. TMT-based proteomic data for 520 regulated proteins in Cluster^#40^ and Cluster^#42^. Protein abundance was corrected by the total peptide amount per sample.**

| **Accession** | **Gene name** | **Control cells** | **LI_72h_ cells** | **MI_72h_ cells** | **HI_72h_ cells** |
| --- | --- | --- | --- | --- | --- |
| P35580 | MYH10 | 36673.73 | 42805.48 | 49911.27 | 66031.91 |
| Q9Y4L1 | HYOU1 | 25204.70 | 25509.33 | 27096.26 | 49493.62 |
| P27797 | CALR | 38218.87 | 43041.90 | 41355.79 | 80783.69 |
| Q9UJZ1 | STOML2 | 9345.80 | 9448.31 | 12168.85 | 20118.08 |
| G3XAI2 | LAMB1 | 5722.92 | 6592.06 | 6484.46 | 8861.70 |
| H0YI37 | ATP5F1B | 332.59 | 499.17 | 544.32 | 1557.59 |
| P04264 | KRT1 | 9136.56 | 12019.57 | 11179.35 | 20957.11 |
| P42166 | TMPO | 6926.57 | 7029.47 | 7280.32 | 7754.96 |
| Q07021 | C1QBP | 3409.31 | 4350.93 | 5549.82 | 10204.73 |
| E9PKU7 | GANAB | 87.80 | 197.58 | 230.91 | 387.41 |
| Q92499 | DDX1 | 5447.68 | 6135.06 | 6366.85 | 7475.80 |
| Q13200 | PSMD2 | 6544.50 | 7210.86 | 6940.22 | 11075.58 |
| Q9HAV7 | GRPEL1 | 9182.92 | 10615.91 | 13462.92 | 24651.36 |
| P07954 | FH | 4763.29 | 5047.54 | 6113.91 | 10210.94 |
| F8W930 | IGF2BP2 | 4936.03 | 5018.65 | 5046.33 | 6200.09 |
| P62195 | PSMC5 | 4295.85 | 4597.07 | 4609.25 | 6547.53 |
| Q14571 | ITPR2 | 4251.90 | 5870.24 | 5327.56 | 8356.93 |
| Q13162 | PRDX4 | 1439.19 | 2214.72 | 2148.89 | 6120.94 |
| Q9UBS4 | DNAJB11 | 3290.28 | 3780.12 | 3830.02 | 7003.03 |
| P30533 | LRPAP1 | 4476.20 | 4809.42 | 4477.05 | 8110.75 |
| Q9BS26 | ERP44 | 3097.13 | 4004.04 | 3711.11 | 7950.31 |
| P05026 | ATP1B1 | 5765.13 | 6114.26 | 6025.46 | 8134.41 |
| Q13724 | MOGS | 4153.99 | 4238.04 | 4409.45 | 4804.68 |
| P63241 | EIF5A | 3945.18 | 6685.78 | 6299.24 | 9421.18 |
| P09104 | ENO2 | 1537.45 | 1591.89 | 1601.33 | 1659.63 |
| P27708 | CAD | 2689.28 | 3234.23 | 3309.14 | 3937.52 |
| P04792 | HSPB1 | 3825.11 | 4361.79 | 4095.06 | 8155.37 |
| P68400 | CSNK2A1 | 2184.90 | 2542.09 | 2699.04 | 4352.67 |
| Q14696 | MESD | 4977.38 | 5204.37 | 5119.55 | 9785.90 |
| O60884 | DNAJA2 | 1534.68 | 1966.52 | 2338.21 | 4126.67 |
| O43242 | PSMD3 | 2339.48 | 2908.00 | 2893.77 | 3621.12 |
| G3V3D1 | NPC2 | 3820.53 | 6148.76 | 5920.05 | 9295.48 |
| P25685 | DNAJB1 | 2533.24 | 4124.45 | 3830.02 | 5627.41 |
| G8JLA2 | MYL6 | 35.98 | 48.70 | 46.66 | 65.38 |
| Q9BSE5 | AGMAT | 1912.43 | 2352.53 | 2288.86 | 3904.93 |
| P21281 | ATP6V1B2 | 2768.34 | 3100.68 | 3176.94 | 5817.34 |
| P36542 | ATP5F1C | 7636.13 | 8857.69 | 9350.26 | 13224.46 |
| Q93084 | ATP2A3 | 246.70 | 531.12 | 493.78 | 1109.46 |
| P45974 | USP5 | 2465.42 | 2646.74 | 2676.36 | 3366.01 |
| Q9BZE9 | ASPSCR1 | 1317.91 | 1437.67 | 1500.67 | 1771.37 |
| Q08380 | LGALS3BP | 1593.76 | 2200.81 | 1862.47 | 4653.75 |
| O95373 | IPO7 | 1206.24 | 1563.19 | 1814.95 | 2941.94 |
| P31689 | DNAJA1 | 1872.12 | 2149.41 | 2345.77 | 3824.42 |
| A0A0C4DFU2 |  | 2801.39 | 4954.32 | 4476.30 | 20295.59 |
| J3KNL6 |  | 1457.78 | 1468.41 | 1536.74 | 1796.98 |
| Q9H0S4 | DDX47 | 1019.66 | 1077.93 | 1196.86 | 1472.62 |
| E7EQY1 | FAM136A | 3185.45 | 3520.84 | 4233.41 | 5781.06 |
| Q9H9B4 | SFXN1 | 1786.74 | 2132.16 | 2154.18 | 2904.11 |
| Q14258 | TRIM25 | 3133.55 | 4301.30 | 4744.36 | 7032.72 |
| D6RER5 | 11-Sep | 2144.16 | 2160.48 | 2443.19 | 3301.02 |
| E9PF82 | CAMK2D | 1138.25 | 1718.96 | 1751.23 | 2683.73 |
| P78540 | ARG2 | 473.33 | 627.46 | 767.56 | 1271.25 |
| Q8N5N7 | MRPL50 | 1683.72 | 1758.15 | 1757.06 | 2540.76 |
| E7EQL5 | DYNC1I2 | 16.61 | 18.46 | 17.17 | 25.61 |
| Q96DZ1 | ERLEC1 | 1622.39 | 2011.39 | 2003.41 | 4742.02 |
| Q13510 | ASAH1 | 1697.82 | 2150.47 | 3217.98 | 5595.02 |
| Q9BT09 | CNPY3 | 2557.89 | 2897.07 | 2920.23 | 5102.66 |
| Q92665 | MRPS31 | 1612.96 | 2087.22 | 2041.64 | 3177.25 |
| A0A087WZM2 | RNASET2 | 2226.68 | 2254.41 | 2285.83 | 3075.02 |
| Q15154 | PCM1 | 1069.23 | 1437.32 | 1518.92 | 2176.04 |
| Q9GZT3 | SLIRP | 2052.12 | 2251.50 | 2785.87 | 6377.79 |
| Q07954 | LRP1 | 2539.81 | 2668.53 | 2813.20 | 3384.05 |
| Q6UN15 | FIP1L1 | 812.84 | 903.43 | 962.61 | 1207.04 |
| Q02543 | RPL18A | 1460.21 | 1619.35 | 1649.92 | 1980.69 |
| O75832 | PSMD10 | 1475.34 | 1816.36 | 1785.36 | 3126.82 |
| Q9NQ50 | MRPL40 | 999.77 | 1230.15 | 1324.63 | 2722.92 |
| X6RAY8 | MRPL4 | 486.13 | 880.21 | 981.29 | 1609.58 |
| Q16718 | NDUFA5 | 1815.29 | 1880.83 | 1996.17 | 3473.10 |
| Q92820 | GGH | 2276.76 | 3557.12 | 3441.76 | 4881.70 |
| P50995 | ANXA11 | 1206.59 | 1601.03 | 1415.89 | 2597.79 |
| Q99805 | TM9SF2 | 813.62 | 1166.82 | 1209.82 | 1826.27 |
| Q53SF7 | COBLL1 | 1391.27 | 1474.80 | 1467.08 | 1908.14 |
| Q9HC07 | TMEM165 | 761.29 | 836.41 | 863.90 | 1074.73 |
| P09417 | QDPR | 1161.18 | 1281.20 | 1204.64 | 2122.31 |
| O00487 | PSMD14 | 2048.49 | 2694.80 | 2638.99 | 3572.81 |
| P01111 | NRAS | 837.67 | 1015.46 | 1120.40 | 1745.38 |
| Q9NQ29 | LUC7L | 245.92 | 319.27 | 295.71 | 406.61 |
| Q86W42 | THOC6 | 715.44 | 773.15 | 823.50 | 997.52 |
| Q9H444 | CHMP4B | 1960.43 | 2010.89 | 1897.35 | 4874.71 |
| Q96RQ3 | MCCC1 | 1155.21 | 1438.45 | 1547.43 | 2054.02 |
| Q15582 | TGFBI | 1269.39 | 1434.33 | 1477.23 | 1808.23 |
| Q8NFH3 | NUP43 | 948.39 | 1021.21 | 1153.88 | 2521.55 |
| Q16186 | ADRM1 | 1852.92 | 1927.40 | 1948.33 | 2035.98 |
| Q8IVS2 | MCAT | 519.00 | 532.19 | 568.41 | 1010.52 |
| E7EVH7 |  | 2013.98 | 2449.15 | 2339.72 | 3538.28 |
| Q5RKV6 | EXOSC6 | 1056.86 | 1242.43 | 1346.77 | 1853.24 |
| Q9BX68 | HINT2 | 620.90 | 623.56 | 780.95 | 1254.18 |
| P02675 | FGB | 989.21 | 1146.30 | 1001.38 | 2197.38 |
| Q13438 | OS9 | 785.94 | 799.56 | 974.70 | 2051.31 |
| Q9Y679 | AUP1 | 2033.53 | 2104.61 | 2138.73 | 2306.80 |
| Q99598 | TSNAX | 1066.28 | 1090.00 | 1089.51 | 1202.77 |
| Q9BYN8 | MRPS26 | 2496.13 | 2789.01 | 2934.70 | 5163.57 |
| Q13098 | GPS1 | 762.32 | 820.50 | 835.06 | 1168.05 |
| Q96P48 | ARAP1 | 800.21 | 915.07 | 896.40 | 1014.21 |
| P46778 | RPL21 | 2963.66 | 3186.52 | 3444.79 | 4348.99 |
| Q96M27 | PRRC1 | 1819.01 | 2147.49 | 2137.55 | 2728.35 |
| P07384 | CAPN1 | 1130.29 | 1287.23 | 1336.94 | 1645.27 |
| Q9UMS0 | NFU1 | 2220.19 | 2694.45 | 3162.90 | 5414.21 |
| E9PLU0 | PDHX | 341.07 | 417.88 | 498.21 | 920.31 |
| O00217 | NDUFS8 | 1494.29 | 2239.43 | 2410.36 | 3620.53 |
| Q9NW13 | RBM28 | 1308.31 | 2068.26 | 2066.70 | 2937.28 |
| Q96HC4 | PDLIM5 | 1067.15 | 1547.36 | 1646.90 | 2958.23 |
| A0A0C4DFZ2 | ARSA | 678.07 | 824.98 | 1012.94 | 2220.28 |
| E9PKF3 | ACAT1 | 363.30 | 483.34 | 509.01 | 1044.28 |
| P20338 | RAB4A | 662.07 | 707.90 | 886.36 | 1633.83 |
| D6RAX7 | COPS4 | 541.32 | 559.66 | 588.28 | 699.93 |
| Q8NHH9 | ATL2 | 1292.48 | 1305.90 | 1452.39 | 1791.74 |
| Q93050 | ATP6V0A1 | 865.35 | 1240.09 | 1467.51 | 2301.37 |
| Q15293 | RCN1 | 1364.62 | 1402.10 | 1748.74 | 2398.17 |
| P56556 | NDUFA6 | 1880.42 | 2797.32 | 3332.47 | 5094.90 |
| P36543 | ATP6V1E1 | 1885.79 | 2030.63 | 2129.01 | 4278.37 |
| Q9BQA1 | WDR77 | 363.99 | 550.36 | 486.11 | 691.79 |
| P30613 | PKLR | 263.22 | 266.24 | 339.95 | 739.65 |
| A0A0D9SFB5 | ADH4 | 72.83 | 159.08 | 172.17 | 190.31 |
| P20073 | ANXA7 | 1200.27 | 1529.90 | 1651.65 | 2581.11 |
| G3V4T2 | PABPN1 | 236.32 | 473.83 | 427.57 | 673.16 |
| P21399 | ACO1 | 1393.60 | 1455.99 | 1303.03 | 2907.02 |
| Q52LJ0 | FAM98B | 717.95 | 858.98 | 786.03 | 1106.16 |
| Q9P287 | BCCIP | 691.65 | 716.42 | 794.24 | 940.68 |
| O60306 | AQR | 1393.51 | 1583.57 | 1700.04 | 2124.44 |
| P20339 | RAB5A | 141.17 | 175.36 | 174.42 | 211.84 |
| Q5T0R9 | CAP1 | 15.48 | 19.81 | 19.01 | 29.68 |
| P21980 | TGM2 | 1567.64 | 1595.35 | 1554.67 | 2081.76 |
| Q9NP58 | ABCB6 | 107.87 | 142.13 | 181.44 | 271.98 |
| P52435 | POLR2J | 780.40 | 999.77 | 964.12 | 1413.06 |
| H7BZ45 | COMT | 208.46 | 277.03 | 342.90 | 512.34 |
| S4R2Z7 | MRPL42 | 271.78 | 330.77 | 394.85 | 1021.97 |
| Q9Y673 | ALG5 | 461.39 | 502.87 | 542.27 | 636.69 |
| Q13868 | EXOSC2 | 910.93 | 1471.39 | 1477.56 | 2229.20 |
| Q92615 | LARP4B | 546.42 | 629.52 | 636.77 | 772.30 |
| Q6P988 | NOTUM | 681.71 | 746.31 | 771.45 | 1716.28 |
| P33316 | DUT | 1301.56 | 1396.99 | 1277.43 | 2117.65 |
| O14841 | OPLAH | 451.62 | 465.81 | 556.09 | 988.79 |
| O60271 | SPAG9 | 747.71 | 985.36 | 986.80 | 1275.71 |
| Q99470 | SDF2 | 514.67 | 517.70 | 568.73 | 1553.32 |
| Q8IY37 | DHX37 | 160.28 | 183.95 | 195.48 | 234.35 |
| H0YAS7 | PABPC1 | 57.70 | 145.90 | 242.57 | 518.36 |
| P14854 | COX6B1 | 979.87 | 1022.77 | 1186.28 | 2580.33 |
| H0YLF3 | B2M | 579.20 | 613.26 | 623.70 | 676.46 |
| Q13085 | ACACA | 288.74 | 322.18 | 320.44 | 425.04 |
| P21549 | AGXT | 452.57 | 491.51 | 467.21 | 630.29 |
| Q16595 | FXN | 195.84 | 270.35 | 346.57 | 1063.29 |
| Q8NBJ5 | COLGALT1 | 519.95 | 973.21 | 892.84 | 1758.37 |
| O96005 | CLPTM1 | 272.22 | 315.51 | 379.51 | 513.89 |
| Q9BU23 | LMF2 | 136.32 | 171.46 | 178.85 | 239.00 |
| Q99523 | SORT1 | 227.84 | 543.69 | 487.19 | 1153.69 |
| Q9Y3B2 | EXOSC1 | 529.38 | 530.77 | 590.01 | 834.18 |
| Q9NRG9 | AAAS | 478.78 | 485.47 | 567.76 | 846.60 |
| Q93008 | USP9X | 404.91 | 414.40 | 420.34 | 649.11 |
| Q7Z4H8 | KDELC2 | 1348.02 | 1633.62 | 1488.46 | 3383.66 |
| H0Y7R8 | AFDN | 312.78 | 363.43 | 370.87 | 599.45 |
| O95302 | FKBP9 | 2082.83 | 2344.36 | 2227.84 | 2867.83 |
| P19440 | GGT1 | 587.85 | 596.37 | 767.67 | 1065.03 |
| P82664 | MRPS10 | 787.24 | 1158.73 | 1415.56 | 2178.76 |
| Q9NPF5 | DMAP1 | 279.91 | 508.97 | 436.21 | 676.27 |
| Q9BZK7 | TBL1XR1 | 465.98 | 714.01 | 693.58 | 1026.23 |
| O75348 | ATP6V1G1 | 754.80 | 800.69 | 712.05 | 1517.43 |
| F8WA42 | SUMF2 | 280.35 | 541.49 | 677.81 | 1188.22 |
| E7EW49 | CLASP2 | 261.92 | 308.76 | 301.75 | 374.22 |
| Q13405 | MRPL49 | 780.83 | 1446.62 | 1741.62 | 2863.56 |
| Q92696 | RABGGTA | 749.69 | 920.32 | 946.73 | 1168.43 |
| A0A0B4J1S4 |  | 288.82 | 490.44 | 651.57 | 1068.91 |
| Q8TF01 | PNISR | 809.29 | 941.76 | 887.33 | 1503.66 |
| O75882 | ATRN | 456.55 | 526.79 | 563.01 | 1002.57 |
| P51003 | PAPOLA | 184.50 | 216.40 | 199.15 | 491.39 |
| P02753 | RBP4 | 1187.64 | 1403.88 | 1394.72 | 2843.58 |
| Q9UHI6 | DDX20 | 309.41 | 326.65 | 374.98 | 464.81 |
| Q9NNW7 | TXNRD2 | 1402.25 | 1449.88 | 1553.16 | 2693.43 |
| Q9HD34 | LYRM4 | 734.13 | 777.90 | 910.55 | 1177.94 |
| O60830 | TIMM17B | 237.10 | 272.48 | 286.42 | 334.25 |
| Q6UXH1 | CRELD2 | 882.39 | 1081.84 | 1114.35 | 1751.00 |
| O75165 | DNAJC13 | 422.55 | 482.77 | 571.00 | 1473.78 |
| Q96NU7 | AMDHD1 | 57.78 | 70.85 | 66.96 | 105.73 |
| Q9NUQ7 | UFSP2 | 547.46 | 562.01 | 586.12 | 768.80 |
| P82932 | MRPS6 | 894.06 | 1208.14 | 1101.50 | 1664.67 |
| P56199 | ITGA1 | 1845.22 | 1915.48 | 1975.65 | 2199.91 |
| Q8IUF8 | RIOX2 | 224.99 | 385.30 | 343.66 | 605.85 |
| P51687 | SUOX | 545.56 | 627.18 | 678.24 | 1161.84 |
| O00764 | PDXK | 96.79 | 132.19 | 120.85 | 176.73 |
| O43813 | LANCL1 | 137.88 | 193.46 | 210.39 | 283.62 |
| P10109 | FDX1 | 286.75 | 307.48 | 323.14 | 695.08 |
| Q9BPX3 | NCAPG | 925.64 | 1085.67 | 1135.63 | 1429.36 |
| Q9GZS3 | WDR61 | 369.70 | 414.69 | 422.28 | 521.85 |
| A0A0B4J1V8 | PPAN-P2RY11 | 249.21 | 259.63 | 260.82 | 352.10 |
| Q9BV38 | WDR18 | 823.65 | 828.74 | 916.60 | 1501.52 |
| Q9NRY4 | ARHGAP35 | 236.75 | 256.30 | 259.09 | 307.29 |
| Q6P087 | RPUSD3 | 454.82 | 593.17 | 738.72 | 1243.90 |
| Q9UHY7 | ENOPH1 | 82.26 | 92.86 | 100.87 | 190.50 |
| Q9HCS7 | XAB2 | 308.98 | 378.69 | 379.84 | 536.20 |
| Q9Y3A2 | UTP11 | 254.05 | 327.86 | 319.68 | 616.52 |
| P30260 | CDC27 | 800.30 | 887.24 | 916.82 | 1781.85 |
| P05161 | ISG15 | 119.28 | 130.06 | 129.17 | 230.27 |
| A0A087WWK8 | IQSEC1 | 78.80 | 106.21 | 96.98 | 132.89 |
| P36954 | POLR2I | 35.81 | 37.63 | 37.48 | 39.38 |
| O75718 | CRTAP | 1160.31 | 1463.59 | 1490.19 | 2690.33 |
| Q2NL82 | TSR1 | 367.45 | 737.86 | 678.03 | 1245.26 |
| Q13042 | CDC16 | 228.36 | 258.92 | 261.25 | 327.08 |
| Q9UID3 | VPS51 | 499.10 | 606.95 | 600.16 | 765.89 |
| O60503 | ADCY9 | 275.68 | 321.61 | 345.60 | 422.33 |
| Q9BWH6 | RPAP1 | 301.54 | 323.03 | 372.71 | 524.18 |
| Q9ULC3 | RAB23 | 457.84 | 528.71 | 545.40 | 975.60 |
| Q9NZJ6 | COQ3 | 54.32 | 116.01 | 144.61 | 259.95 |
| O94903 | PLPBP | 539.93 | 542.41 | 635.58 | 830.11 |
| P57081 | WDR4 | 542.61 | 890.65 | 1016.29 | 1605.12 |
| Q15648 | MED1 | 456.98 | 602.76 | 640.44 | 1184.53 |
| Q9H7L9 | SUDS3 | 471.86 | 613.26 | 609.66 | 852.03 |
| O00743 | PPP6C | 86.50 | 93.71 | 93.42 | 174.98 |
| P23258 | TUBG1 | 206.82 | 213.49 | 215.89 | 269.27 |
| O75312 | ZPR1 | 292.28 | 343.34 | 366.01 | 460.16 |
| Q9UP83 | COG5 | 254.22 | 279.72 | 269.79 | 316.21 |
| Q6UXN9 | WDR82 | 410.44 | 514.65 | 551.77 | 824.87 |
| Q8WW59 | SPRYD4 | 581.63 | 806.59 | 801.04 | 1398.90 |
| Q8IWZ8 | SUGP1 | 158.21 | 158.82 | 175.50 | 290.80 |
| P52735 | VAV2 | 296.78 | 297.83 | 329.73 | 416.12 |
| O43765 | SGTA | 297.13 | 342.49 | 325.30 | 386.05 |
| Q9Y6D6 | ARFGEF1 | 311.57 | 332.83 | 321.30 | 448.32 |
| P08397 | HMBS | 92.55 | 105.07 | 103.14 | 148.99 |
| Q9NXH9 | TRMT1 | 205.18 | 295.06 | 313.53 | 429.31 |
| F2Z2X4 | XPO4 | 186.58 | 188.07 | 194.18 | 233.57 |
| Q53LP3 | SOWAHC | 245.57 | 351.22 | 360.18 | 501.09 |
| Q4KMQ2 | ANO6 | 833.60 | 870.48 | 917.25 | 1570.39 |
| Q9Y223 | GNE | 404.82 | 453.67 | 518.62 | 660.17 |
| Q96PE7 | MCEE | 532.23 | 740.06 | 775.01 | 1478.05 |
| O75027 | ABCB7 | 290.73 | 317.64 | 371.31 | 494.69 |
| P00488 | F13A1 | 93.42 | 108.06 | 148.61 | 802.95 |
| Q4V339 | CBWD6 | 176.63 | 197.72 | 248.29 | 572.67 |
| Q9NWU2 | GID8 | 344.53 | 349.66 | 416.13 | 676.46 |
| P00738 | HP | 306.30 | 372.52 | 334.37 | 524.37 |
| P35625 | TIMP3 | 129.75 | 263.32 | 220.00 | 491.20 |
| Q86VR2 | RETREG3 | 241.33 | 423.49 | 428.76 | 695.67 |
| Q6UWM9 | UGT2A3 | 207.17 | 256.08 | 270.00 | 334.25 |
| Q9Y4C2 | TCAF1 | 465.37 | 641.10 | 830.09 | 2418.54 |
| P46976 | GYG1 | 360.88 | 401.63 | 439.56 | 591.10 |
| P61758 | VBP1 | 205.70 | 264.74 | 237.71 | 308.06 |
| O95169 | NDUFB8 | 158.21 | 214.20 | 206.93 | 291.77 |
| O95372 | LYPLA2 | 967.42 | 1118.54 | 1127.85 | 1292.20 |
| Q8NAV1 | PRPF38A | 263.82 | 334.68 | 320.98 | 465.39 |
| Q9Y2S2 | CRYL1 | 59.43 | 63.40 | 58.86 | 87.30 |
| Q15750 | TAB1 | 50.08 | 73.76 | 71.28 | 103.40 |
| O60888 | CUTA | 465.89 | 494.56 | 491.62 | 685.97 |
| E7ETH0 | CFI | 84.77 | 102.23 | 85.32 | 187.79 |
| K7ESQ2 | SPC24 | 48.35 | 52.25 | 54.76 | 78.76 |
| Q8NFF5 | FLAD1 | 101.29 | 103.94 | 123.44 | 240.55 |
| Q9H299 | SH3BGRL3 | 118.76 | 140.22 | 143.64 | 170.91 |
| A0A0C4DFX3 |  | 132.60 | 139.58 | 140.72 | 153.64 |
| A0A0C4DGI2 | NXN | 45.84 | 73.91 | 70.52 | 113.49 |
| P07358 | C8B | 276.97 | 282.35 | 289.12 | 956.98 |
| Q6ZXV5 | TMTC3 | 285.02 | 305.50 | 423.36 | 753.28 |
| Q9BY89 | KIAA1671 | 296.44 | 356.76 | 387.51 | 649.88 |
| Q0VDF9 | HSPA14 | 206.82 | 242.52 | 238.36 | 288.28 |
| P51797 | CLCN6 | 207.17 | 215.05 | 219.24 | 246.96 |
| Q9NRG4 | SMYD2 | 149.90 | 168.83 | 164.38 | 226.78 |
| Q9BWJ5 | SF3B5 | 165.47 | 179.76 | 186.62 | 257.24 |
| P04003 | C4BPA | 95.15 | 102.23 | 111.78 | 238.23 |
| Q8IVP5 | FUNDC1 | 44.11 | 67.09 | 68.04 | 95.45 |
| Q96SU4 | OSBPL9 | 274.46 | 283.13 | 287.93 | 351.13 |
| Q9H0T7 | RAB17 | 214.69 | 298.04 | 382.32 | 1399.68 |
| O43157 | PLXNB1 | 96.97 | 118.92 | 150.66 | 261.12 |
| Q9HBM1 | SPC25 | 481.72 | 581.39 | 514.73 | 842.13 |
| Q9NVX2 | NLE1 | 169.63 | 212.14 | 208.87 | 387.41 |
| Q9BV20 | MRI1 | 228.19 | 250.40 | 254.13 | 362.38 |
| Q96T51 | RUFY1 | 55.53 | 76.32 | 96.01 | 166.64 |
| Q96GM8 | TOE1 | 49.39 | 59.49 | 72.36 | 142.00 |
| Q9NZJ9 | NUDT4 | 10.90 | 15.34 | 15.88 | 22.70 |
| Q8IZ73 | RPUSD2 | 286.06 | 304.86 | 297.22 | 408.36 |
| Q8IZ81 | ELMOD2 | 158.90 | 164.21 | 197.53 | 342.98 |
| Q9NZN8 | CNOT2 | 840.95 | 857.07 | 871.24 | 1056.89 |
| P00374 | DHFR | 151.03 | 230.31 | 223.24 | 417.09 |
| Q7Z6B0 | CCDC91 | 1080.12 | 1437.32 | 2568.15 | 5218.67 |
| P56937 | HSD17B7 | 426.62 | 476.03 | 466.89 | 691.79 |
| Q9Y6K9 | IKBKG | 214.61 | 223.50 | 239.55 | 497.99 |
| O95235 | KIF20A | 80.79 | 101.03 | 101.30 | 136.18 |
| Q9P2K3 | RCOR3 | 80.36 | 89.81 | 107.57 | 177.51 |
| F5H0C4 | PAAF1 | 154.58 | 172.09 | 165.89 | 269.27 |
| Q99547 | MPHOSPH6 | 294.53 | 371.59 | 379.95 | 496.82 |
| Q5T2T1 | MPP7 | 301.71 | 405.96 | 365.91 | 480.33 |
| Q52LW3 | ARHGAP29 | 251.80 | 282.00 | 312.66 | 566.08 |
| Q96CN9 | GCC1 | 94.72 | 162.44 | 174.20 | 375.19 |
| A0A087X1E4 | ARFIP2 | 37.63 | 43.73 | 40.93 | 48.50 |
| Q9NV88 | INTS9 | 29.84 | 41.75 | 42.34 | 64.41 |
| Q9NWT8 | AURKAIP1 | 519.43 | 580.54 | 658.37 | 1001.79 |
| Q71F23 | CENPU | 156.74 | 187.86 | 181.98 | 246.18 |
| Q9Y287 | ITM2B | 26.30 | 45.37 | 37.58 | 57.23 |
| A0A1B0GTL5 | RAB11FIP5 | 156.13 | 179.62 | 168.16 | 215.14 |
| O75376 | NCOR1 | 149.82 | 157.26 | 188.68 | 323.78 |
| Q16864 | ATP6V1F | 288.74 | 357.96 | 419.58 | 1983.60 |
| P30291 | WEE1 | 83.13 | 98.40 | 96.98 | 123.57 |
| Q10472 | GALNT1 | 74.04 | 197.87 | 180.58 | 436.30 |
| Q13416 | ORC2 | 444.96 | 491.44 | 515.70 | 1925.40 |
| Q86WU2 | LDHD | 69.63 | 101.74 | 96.44 | 165.48 |
| Q8NEB9 | PIK3C3 | 59.68 | 97.97 | 121.72 | 227.36 |
| Q8NEH6 | MNS1 | 300.24 | 602.76 | 689.80 | 3099.07 |
| Q9UEU0 | VTI1B | 41.61 | 42.88 | 49.36 | 89.82 |
| Q8IUR0 | TRAPPC5 | 155.53 | 292.43 | 256.07 | 438.82 |
| Q99674 | CGREF1 | 177.84 | 301.02 | 463.43 | 2215.23 |
| Q01459 | CTBS | 241.33 | 252.82 | 231.23 | 577.33 |
| Q8WUW1 | BRK1 | 276.71 | 294.49 | 298.62 | 588.97 |
| A0A0A0MR39 | MYEF2 | 229.48 | 573.08 | 822.53 | 2459.47 |
| Q02083 | NAAA | 51.90 | 70.00 | 77.11 | 110.00 |
| D6RF75 | GFM2 | 477.13 | 717.56 | 920.16 | 3657.59 |
| Q56P03 | EAPP | 104.32 | 201.77 | 163.73 | 284.20 |
| O94923 | GLCE | 126.64 | 169.18 | 162.00 | 331.15 |
| Q9BX93 | PLA2G12B | 149.73 | 240.32 | 241.92 | 503.03 |
| B4E1Z4 |  | 379.48 | 490.80 | 545.73 | 830.11 |
| O75461 | E2F6 | 33.39 | 39.90 | 49.46 | 70.61 |
| O75529 | TAF5L | 62.19 | 68.80 | 65.23 | 82.25 |
| Q9NP66 | HMG20A | 256.47 | 300.60 | 381.67 | 919.73 |
| Q13469 | NFATC2 | 92.38 | 158.04 | 153.79 | 265.97 |
| P78562 | PHEX | 241.08 | 495.34 | 716.37 | 1415.20 |
| Q7Z4G4 | TRMT11 | 107.17 | 163.58 | 223.67 | 475.68 |
| P04156 | PRNP | 156.56 | 245.29 | 253.69 | 488.48 |
| Q93088 | BHMT | 229.48 | 342.20 | 280.69 | 571.70 |
| Q13315 | ATM | 268.24 | 324.17 | 538.49 | 1374.07 |
| K7ERV3 | TK1 | 99.99 | 101.52 | 106.60 | 227.75 |
| Q86UA1 | PRPF39 | 111.50 | 138.58 | 132.62 | 160.82 |
| P36639 | NUDT1 | 139.52 | 188.78 | 171.29 | 234.15 |
| P09669 | COX6C | 357.42 | 378.76 | 482.33 | 686.36 |
| Q99611 | SEPHS2 | 222.56 | 236.91 | 226.26 | 292.16 |
| Q92729 | PTPRU | 45.76 | 112.74 | 175.18 | 765.12 |
| Q69YL0 | NCBP2-AS2 | 300.15 | 507.98 | 787.65 | 1547.89 |
| A0A0D9SF50 | BRCC3 | 177.15 | 183.38 | 179.71 | 324.94 |
| O75146 | HIP1R | 178.10 | 218.95 | 297.00 | 496.43 |
| Q86T03 | PIP4P1 | 121.19 | 156.62 | 207.79 | 432.42 |
| P37802 | TAGLN2 | 289.26 | 472.98 | 418.29 | 1230.90 |
| A0A0A0MT71 | C16orf89 | 270.83 | 619.23 | 632.88 | 1175.42 |
| P56589 | PEX3 | 76.81 | 93.57 | 100.12 | 126.29 |
| Q3BDU5 | LMNA | 161.75 | 256.44 | 381.57 | 404.09 |
| Q9UQ35 | SRRM2 | 7095.33 | 7977.20 | 8943.63 | 9313.91 |
| P55084 | HADHB | 7737.16 | 10102.18 | 11478.84 | 12936.38 |
| Q7Z406 | MYH14 | 1774.55 | 2028.50 | 2464.14 | 2656.57 |
| Q8TCS8 | PNPT1 | 5710.47 | 7042.39 | 8958.54 | 9059.58 |
| O95831 | AIFM1 | 4192.65 | 4663.66 | 5485.35 | 5655.93 |
| P49756 | RBM25 | 7457.59 | 8493.98 | 9286.97 | 10557.61 |
| Q99497 | PARK7 | 6732.38 | 8439.59 | 9320.99 | 10324.04 |
| P43686 | PSMC4 | 3605.75 | 3869.50 | 3984.79 | 4328.42 |
| Q86TX2 | ACOT1 | 3017.90 | 3571.32 | 3924.63 | 3961.77 |
| P47985 | UQCRFS1 | 3660.76 | 3758.68 | 4170.66 | 4880.14 |
| Q9UIJ7 | AK3 | 5493.96 | 6469.88 | 8434.63 | 11782.49 |
| P17301 | ITGA2 | 2012.51 | 2036.45 | 2672.91 | 3315.77 |
| P23229 | ITGA6 | 3629.88 | 4579.46 | 5300.78 | 6146.74 |
| O94905 | ERLIN2 | 4703.43 | 4973.28 | 5621.64 | 5817.14 |
| E9PF18 | HADH | 7670.99 | 7798.29 | 8799.99 | 9673.38 |
| C9JIZ6 | PSAP | 6830.99 | 7037.70 | 7573.97 | 7841.09 |
| P51149 | RAB7A | 3955.64 | 4220.08 | 4373.05 | 4460.14 |
| O75947 | ATP5PD | 5531.33 | 6259.66 | 7875.51 | 9605.48 |
| E7EX59 | PCCB | 2481.51 | 2907.65 | 3300.71 | 3889.41 |
| Q99856 | ARID3A | 1391.96 | 2179.44 | 2914.29 | 3375.71 |
| P16989 | YBX3 | 419.61 | 546.60 | 662.26 | 803.92 |
| O75955 | FLOT1 | 1971.16 | 2210.61 | 2394.37 | 2728.35 |
| Q96GQ7 | DDX27 | 3464.84 | 4634.27 | 5382.75 | 5585.90 |
| P82650 | MRPS22 | 2214.92 | 3840.04 | 4810.78 | 5832.47 |
| Q8NBQ5 | HSD17B11 | 3352.91 | 3978.91 | 4405.77 | 5065.22 |
| E7EPT4 | NDUFV2 | 3322.64 | 3958.82 | 4704.07 | 4949.59 |
| P18031 | PTPN1 | 1026.93 | 1644.84 | 1939.58 | 2690.91 |
| C9JFV4 | PELP1 | 1100.80 | 1208.21 | 1415.02 | 1619.47 |
| O75489 | NDUFS3 | 3892.41 | 4183.38 | 4432.45 | 4456.46 |
| P21912 | SDHB | 5273.73 | 5515.62 | 7233.77 | 9261.14 |
| P22413 | ENPP1 | 1375.95 | 1483.11 | 1786.76 | 2214.07 |
| Q96EY1 | DNAJA3 | 2028.51 | 2196.98 | 2671.50 | 2856.97 |
| O14974 | PPP1R12A | 1615.21 | 1862.94 | 2046.61 | 2065.47 |
| Q9H3G5 | CPVL | 3858.59 | 4103.51 | 4953.01 | 5580.47 |
| Q8WWY3 | PRPF31 | 1817.88 | 1883.67 | 2122.53 | 2284.10 |
| Q9BPW8 | NIPSNAP1 | 4321.10 | 5453.50 | 6399.36 | 6478.66 |
| J3QTA6 | CHCHD6 | 1742.02 | 1964.96 | 2275.57 | 2921.76 |
| Q9UNW1 | MINPP1 | 969.66 | 1698.74 | 2266.69 | 2383.81 |
| Q8WUD1 | RAB2B | 111.07 | 125.45 | 172.69 | 252.58 |
| P51114 | FXR1 | 1849.63 | 2022.40 | 2159.04 | 2325.61 |
| O95757 | HSPA4L | 423.16 | 533.18 | 587.85 | 660.36 |
| Q9NUB1 | ACSS1 | 1849.94 | 2085.94 | 2244.41 | 2289.30 |
| Q8WYP5 | AHCTF1 | 683.52 | 862.89 | 1000.73 | 1003.93 |
| P78406 | RAE1 | 1500.17 | 1546.37 | 2139.71 | 3028.07 |
| H0Y8P4 | UTP15 | 2647.94 | 3066.89 | 3439.71 | 3612.00 |
| Q9Y2S7 | POLDIP2 | 1941.06 | 2405.49 | 2789.65 | 3435.85 |
| Q8N0X4 | CLYBL | 470.04 | 615.89 | 734.51 | 924.78 |
| Q92769 | HDAC2 | 1450.43 | 1530.04 | 1688.91 | 1724.62 |
| F8VRH0 | PCBP2 | 506.11 | 522.46 | 677.81 | 841.55 |
| Q01780 | EXOSC10 | 1202.78 | 1450.45 | 1588.58 | 1668.75 |
| Q9Y6M5 | SLC30A1 | 988.52 | 1321.59 | 1508.44 | 1747.51 |
| J3KNN5 | DDX41 | 1115.50 | 1278.78 | 1582.75 | 1757.40 |
| Q96DA6 | DNAJC19 | 1444.20 | 1995.06 | 2583.16 | 3586.00 |
| P49189 | ALDH9A1 | 1569.20 | 1712.80 | 2053.44 | 2309.70 |
| Q96RS6 | NUDCD1 | 1053.05 | 1284.25 | 1374.31 | 1539.74 |
| Q9UBC2 | EPS15L1 | 410.79 | 551.28 | 591.95 | 745.72 |
| P05165 | PCCA | 1215.93 | 1693.04 | 1817.54 | 2279.44 |
| Q15007 | WTAP | 823.91 | 918.19 | 1077.63 | 1355.83 |
| P11234 | RALB | 1988.55 | 2164.46 | 2419.86 | 2541.53 |
| Q8TDI0 | CHD5 | 171.62 | 188.78 | 209.09 | 223.68 |
| O75891 | ALDH1L1 | 484.92 | 497.75 | 542.92 | 560.65 |
| Q9H9Q2 | COPS7B | 676.17 | 722.46 | 822.96 | 999.46 |
| Q96A35 | MRPL24 | 1891.58 | 2312.63 | 2584.99 | 3209.07 |
| Q99615 | DNAJC7 | 1093.19 | 1304.84 | 1421.94 | 1467.57 |
| Q9H6Z4 | RANBP3 | 1687.87 | 1921.01 | 2021.99 | 2342.10 |
| P62072 | TIMM10 | 1414.19 | 1656.06 | 2565.55 | 3121.38 |
| Q13443 | ADAM9 | 918.11 | 1031.64 | 1102.25 | 1184.92 |
| Q8WTT2 | NOC3L | 1301.31 | 1598.19 | 1957.94 | 2348.51 |
| Q9NVS9 | PNPO | 1118.27 | 1161.64 | 1375.60 | 1564.38 |
| Q5T1J5 | CHCHD2P9 | 650.31 | 786.35 | 869.51 | 1066.00 |
| P19525 | EIF2AK2 | 1181.07 | 1282.62 | 1400.66 | 1422.96 |
| Q9NXV6 | CDKN2AIP | 194.71 | 264.89 | 392.26 | 508.07 |
| H0Y2S1 |  | 612.25 | 684.05 | 935.72 | 1050.87 |
| Q14141 | 6-Sep | 238.74 | 246.29 | 255.75 | 255.49 |
| Q9Y5J7 | TIMM9 | 974.85 | 1253.44 | 1554.78 | 1845.28 |
| Q9UBU6 | FAM8A1 | 83.39 | 126.02 | 174.10 | 167.81 |
| Q92643 | PIGK | 462.34 | 569.89 | 619.49 | 745.91 |
| Q9BQ52 | ELAC2 | 770.80 | 855.08 | 891.33 | 949.80 |
| Q9HA77 | CARS2 | 815.87 | 982.52 | 1047.07 | 1188.03 |
| O15126 | SCAMP1 | 1543.16 | 1607.64 | 1656.19 | 1772.53 |
| Q9P0S9 | TMEM14C | 128.28 | 150.94 | 220.11 | 251.42 |
| P82663 | MRPS25 | 926.67 | 1704.90 | 2211.64 | 2264.51 |
| Q04323 | UBXN1 | 157.86 | 214.98 | 304.67 | 303.60 |
| O00186 | STXBP3 | 351.80 | 433.93 | 510.19 | 576.55 |
| Q9Y375 | NDUFAF1 | 303.96 | 430.24 | 560.52 | 633.39 |
| Q9Y646 | CPQ | 148.69 | 195.31 | 231.45 | 288.28 |
| O75600 | GCAT | 236.14 | 282.49 | 331.99 | 331.54 |
| Q9NR28 | DIABLO | 372.30 | 369.75 | 478.87 | 552.50 |
| Q8TDD1 | DDX54 | 824.78 | 1074.03 | 1251.08 | 1276.29 |
| B8ZZA8 | GLS | 407.85 | 530.63 | 621.44 | 690.62 |
| Q9BRT2 | UQCC2 | 524.71 | 962.35 | 1131.85 | 1563.80 |
| Q9HC36 | MRM3 | 386.74 | 401.98 | 524.34 | 648.33 |
| Q9P275 | USP36 | 170.92 | 193.61 | 231.66 | 240.75 |
| P25325 | MPST | 456.11 | 488.88 | 523.37 | 568.41 |
| P61225 | RAP2B | 566.92 | 752.42 | 880.96 | 1160.09 |
| O94832 | MYO1D | 592.78 | 640.74 | 686.67 | 730.59 |
| Q15006 | EMC2 | 346.61 | 382.74 | 487.08 | 663.46 |
| Q9Y5L4 | TIMM13 | 201.03 | 207.45 | 255.75 | 312.53 |
| H0YGM0 | CLPB | 276.37 | 367.69 | 406.73 | 494.69 |
| Q12929 | EPS8 | 270.74 | 289.03 | 378.22 | 426.60 |
| P13640 | MT1G | 53.89 | 70.57 | 82.84 | 83.22 |
| Q9BVS5 | TRMT61B | 513.72 | 597.01 | 624.57 | 725.54 |
| Q12893 | TMEM115 | 219.62 | 266.38 | 374.44 | 564.14 |
| O95777 | LSM8 | 171.62 | 246.50 | 292.03 | 340.46 |
| O95639 | CPSF4 | 274.90 | 459.98 | 505.23 | 673.55 |
| P50851 | LRBA | 250.68 | 325.73 | 367.96 | 458.99 |
| Q9Y6G9 | DYNC1LI1 | 465.28 | 549.08 | 624.35 | 726.71 |
| Q92917 | GPKOW | 418.14 | 490.09 | 523.91 | 564.72 |
| B1AN86 | TJP2 | 17.47 | 20.45 | 28.40 | 35.11 |
| Q8WWV3 | RTN4IP1 | 673.58 | 684.76 | 705.57 | 718.75 |
| Q9BST9 | RTKN | 703.59 | 1022.20 | 1522.92 | 1849.36 |
| O60443 | GSDME | 464.16 | 641.52 | 813.24 | 853.58 |
| Q92925 | SMARCD2 | 165.47 | 193.11 | 218.59 | 251.42 |
| Q99496 | RNF2 | 277.32 | 320.19 | 383.62 | 419.03 |
| Q7Z5J4 | RAI1 | 152.59 | 187.29 | 230.47 | 237.26 |
| O15226 | NKRF | 1322.58 | 1409.84 | 1497.21 | 1671.85 |
| Q9H2M9 | RAB3GAP2 | 119.11 | 141.21 | 167.94 | 174.40 |
| Q53F19 | NCBP3 | 438.12 | 451.68 | 527.58 | 573.26 |
| Q5TDH0 | DDI2 | 333.63 | 421.36 | 445.83 | 540.86 |
| P14859 | POU2F1 | 284.58 | 294.99 | 313.63 | 323.39 |
| E9PNV3 | RDX | 147.31 | 150.65 | 160.27 | 177.70 |
| A0A140TA86 | MICOS13 | 228.10 | 334.68 | 388.80 | 520.30 |
| P41208 | CETN2 | 119.37 | 177.06 | 217.62 | 247.93 |
| Q13620 | CUL4B | 222.13 | 288.10 | 365.47 | 491.39 |
| P49770 | EIF2B2 | 304.83 | 324.31 | 363.96 | 374.99 |
| H0Y5K5 | ERGIC3 | 191.34 | 414.76 | 486.11 | 676.07 |
| Q969S9 | GFM2 | 503.26 | 485.97 | 646.82 | 763.57 |
| Q9P0K7 | RAI14 | 489.50 | 580.89 | 721.55 | 912.94 |
| Q96E11 | MRRF | 438.38 | 529.77 | 586.66 | 600.22 |
| H0Y2M6 | CLCN7 | 110.46 | 131.77 | 239.76 | 385.47 |
| Q8IXM3 | MRPL41 | 675.65 | 732.11 | 1006.24 | 1475.33 |
| Q8TC07 | TBC1D15 | 391.41 | 405.39 | 460.62 | 504.97 |
| Q7L5N7 | LPCAT2 | 393.32 | 413.69 | 562.14 | 772.49 |
| P05543 | SERPINA7 | 50.78 | 80.72 | 100.01 | 122.02 |
| P28290 | ITPRID2 | 540.80 | 592.89 | 667.88 | 733.30 |
| Q96HN2 | AHCYL2 | 347.04 | 406.38 | 454.57 | 474.90 |
| P12429 | ANXA3 | 126.64 | 149.87 | 177.34 | 211.26 |
| P51553 | IDH3G | 225.51 | 399.35 | 599.51 | 592.66 |
| Q7L5N1 | COPS6 | 200.51 | 240.39 | 256.39 | 293.71 |
| Q8TCC3 | MRPL30 | 427.14 | 599.35 | 680.08 | 760.85 |
| Q9Y508 | RNF114 | 344.36 | 442.16 | 536.55 | 632.42 |
| Q9BSF4 | TIMM29 | 59.86 | 87.68 | 109.73 | 123.96 |
| P21675 | TAF1 | 38.84 | 47.21 | 68.80 | 82.64 |
| E7END6 | PROC | 75.43 | 90.95 | 104.98 | 112.52 |
| O15381 | NVL | 128.37 | 137.24 | 150.12 | 153.06 |
| Q8NFH4 | NUP37 | 110.29 | 113.10 | 135.76 | 164.51 |
| Q86UF2 | CTAGE6 | 260.45 | 314.51 | 479.74 | 599.83 |
| Q15650 | TRIP4 | 363.21 | 454.38 | 563.55 | 787.81 |
| Q8IXB1 | DNAJC10 | 333.46 | 486.32 | 579.64 | 696.83 |
| O14976 | GAK | 189.00 | 234.93 | 268.49 | 282.46 |
| Q9H7D7 | WDR26 | 120.41 | 136.31 | 146.12 | 172.27 |
| Q8NBN3 | TMEM87A | 1104.60 | 1257.91 | 1505.53 | 1831.32 |
| Q5TDF0 | NTPCR | 137.97 | 182.46 | 204.77 | 253.36 |
| Q96EK6 | GNPNAT1 | 97.31 | 96.84 | 109.62 | 120.47 |
| Q9Y5L3 | ENTPD2 | 48.96 | 50.98 | 63.72 | 72.75 |
| Q9H6T3 | RPAP3 | 47.23 | 52.40 | 64.69 | 73.72 |
| Q8N0X7 | SPART | 221.70 | 231.45 | 256.07 | 284.40 |
| Q8N8R5 | C2orf69 | 79.67 | 130.70 | 163.94 | 197.88 |
| Q9BYD1 | MRPL13 | 859.12 | 997.64 | 1123.64 | 1248.17 |
| J3KR72 | TAF6 | 204.05 | 240.46 | 273.35 | 300.89 |
| Q9Y2Z0 | SUGT1 | 298.68 | 341.49 | 365.80 | 407.97 |
| Q9UMZ2 | SYNRG | 264.78 | 370.95 | 507.39 | 719.72 |
| Q6P4Q7 | CNNM4 | 37.89 | 63.54 | 79.92 | 117.56 |
| O94887 | FARP2 | 139.09 | 224.06 | 278.10 | 302.05 |
| P08047 | SP1 | 72.75 | 92.58 | 121.28 | 161.99 |
| O43301 | HSPA12A | 295.05 | 295.84 | 301.32 | 304.57 |
| Q8ND76 | CCNY | 30.53 | 46.08 | 56.59 | 68.87 |
| Q9BUT9 | MCRIP2 | 40.05 | 47.14 | 67.93 | 95.45 |
| O00754 | MAN2B1 | 280.17 | 389.63 | 509.33 | 559.87 |
| Q8N6H7 | ARFGAP2 | 151.98 | 221.15 | 298.62 | 405.06 |
| A6NDE4 | RBMY1B | 45.33 | 47.00 | 59.40 | 67.70 |
| A0A087WYL7 | MED6 | 198.69 | 382.81 | 472.93 | 598.67 |
| A0A0C4DFN1 |  | 49.91 | 56.16 | 62.64 | 75.66 |
| O75175 | CNOT3 | 235.97 | 271.92 | 331.24 | 445.80 |
| Q96DA2 | RAB39B | 437.95 | 748.58 | 974.92 | 1323.05 |
| Q7Z7N9 | TMEM179B | 80.10 | 90.02 | 97.42 | 97.19 |
| P04150 | NR3C1 | 100.95 | 112.24 | 136.73 | 167.03 |
| Q2NKX8 | ERCC6L | 109.85 | 152.00 | 224.53 | 351.13 |
| O96011 | PEX11B | 179.66 | 220.44 | 251.43 | 280.13 |
| P32519 | ELF1 | 167.03 | 203.40 | 217.95 | 266.16 |
| D6REA1 | SIL1 | 126.38 | 175.43 | 363.75 | 651.82 |
| Q96NT5 | SLC46A1 | 110.37 | 143.27 | 190.62 | 215.92 |
| P41236 | PPP1R2 | 524.88 | 647.41 | 740.45 | 785.87 |
| H0YH36 | SLC3A2 | 262.01 | 299.11 | 358.99 | 452.98 |
| Q86YS7 | C2CD5 | 125.68 | 144.62 | 177.98 | 189.53 |
| J3QR07 | YTHDC1 | 321.35 | 335.10 | 355.54 | 372.66 |
| Q9NZJ5 | EIF2AK3 | 120.75 | 142.92 | 174.96 | 211.45 |
| Q9H2F3 | HSD3B7 | 221.79 | 282.35 | 454.14 | 575.97 |
| Q567U6 | CCDC93 | 201.89 | 251.18 | 278.75 | 323.00 |
| M0R1Y2 | KDELR1 | 205.61 | 319.27 | 404.79 | 614.77 |
| O95396 | MOCS3 | 95.06 | 99.32 | 127.22 | 142.39 |
| Q96DT7 | ZBTB10 | 444.70 | 748.58 | 943.17 | 1020.22 |
| H0Y9Z5 | CNOT6L | 49.05 | 58.29 | 74.84 | 88.85 |
