# Supplementary material for: Mitochondrial metabolic study guided by proteomics analysis in hepatocellular carcinoma cells surviving long-term incubation with the highest dose of sorafenib
Source: Aging (Albany NY). 2019 Dec 26;11(24):12452–75. doi: 10.18632/aging.102582 (PMC6949094; doi:10.18632/aging.102582)
Supplement: Supplementary Table 2 [file aging-11-102582-s001..docx]

**Supplementary Table 2. Functional annotation and statistical analysis for the nodes in Figure 2B.**

| **GO-term ‘cellular compartment’** | **p-value** | **Benjamini-Hochberg corrected p-value (Cp-value)** | **cluster frequency**  **(%)** | **total frequency (%)** |
| --- | --- | --- | --- | --- |
| mitochondrion | 1.69E-77 | 2.75E-75 | 87.1% | 7.7% |
| mitochondrial part | 3.64E-38 | 2.97E-36 | 47.3% | 3.7% |
| cytoplasmic part | 2.55E-35 | 1.39E-33 | 92.4% | 31.4% |
| cytoplasm | 6.95E-25 | 2.83E-23 | 95.6% | 46.6% |
| mitochondrial envelope | 3.25E-22 | 1.06E-20 | 30.1% | 2.6% |
| mitochondrial matrix | 1.21E-21 | 2.81E-20 | 23.6% | 1.3% |
| mitochondrial lumen | 1.21E-21 | 2.81E-20 | 23.6% | 1.3% |
| mitochondrial inner membrane | 2.45E-21 | 4.99E-20 | 25.8% | 1.8% |
| organelle inner membrane | 2.20E-20 | 3.99E-19 | 25.8% | 1.9% |
| mitochondrial membrane | 3.20E-19 | 5.21E-18 | 26.8% | 2.5% |
| intracellular membrane-bounded organelle | 3.95E-18 | 5.73E-17 | 92.4% | 50.9% |
| membrane-bounded organelle | 4.22E-18 | 5.73E-17 | 92.4% | 51.0% |
| organelle envelope | 1.92E-17 | 2.41E-16 | 30.1% | 4.0% |
| envelope | 3.19E-17 | 3.71E-16 | 30.1% | 4.1% |
| intracellular organelle | 2.09E-16 | 2.27E-15 | 94.6% | 56.9% |
| organelle | 2.36E-16 | 2.41E-15 | 94.6% | 57.0% |
| intracellular part | 8.67E-13 | 8.31E-12 | 96.7% | 66.8% |
| mitochondrial membrane part | 9.95E-12 | 9.01E-11 | 12.9% | 0.8% |
| intracellular | 1.26E-11 | 1.08E-10 | 96.7% | 69.0% |
| membrane-enclosed lumen | 4.74E-10 | 3.87E-09 | 34.4% | 10.4% |
| mitochondrial intermembrane space | 7.21E-10 | 5.60E-09 | 7.5% | 0.2% |
| ribosome | 1.11E-09 | 8.20E-09 | 12.9% | 1.1% |
| organelle envelope lumen | 2.43E-09 | 1.72E-08 | 7.5% | 0.2% |
| organellar ribosome | 3.28E-07 | 2.14E-06 | 6.4% | 0.2% |
| mitochondrial ribosome | 3.28E-07 | 2.14E-06 | 6.4% | 0.2% |
| nucleoid | 8.03E-07 | 4.85E-06 | 5.3% | 0.1% |
| mitochondrial nucleoid | 8.03E-07 | 4.85E-06 | 5.3% | 0.1% |
| mitochondrial respiratory chain | 2.80E-06 | 1.53E-05 | 6.4% | 0.4% |
| organellar small ribosomal subunit | 2.81E-06 | 1.53E-05 | 4.3% | 0.1% |
| mitochondrial small ribosomal subunit | 2.81E-06 | 1.53E-05 | 4.3% | 0.1% |
| organelle membrane | 3.12E-06 | 1.62E-05 | 29.0% | 11.4% |
| intracellular organelle lumen | 3.17E-06 | 1.62E-05 | 26.8% | 10.0% |
| mitochondrial intermembrane space protein transporter complex | 3.51E-06 | 1.73E-05 | 3.2% | 0.0% |
| organelle lunen | 4.63E-06 | 2.22E-05 | 26.8% | 10.2% |
| respiratory chain | 4.90E-06 | 2.28E-05 | 6.4% | 0.4% |
| intracellular organelle part | 5.13E-06 | 2.32E-05 | 51.6% | 29.3% |
| organelle part | 7.79E-06 | 3.43E-05 | 51.6% | 29.7% |
| ribonucleoprotein complex | 2.70E-05 | 1.16E-04 | 12.9% | 3.0% |
| fatty acid beta-oxidation multienzyme complex | 3.19E-05 | 1.33E-04 | 2.1% | 0.0% |
| mitochondrial inner membrane presquence translocase complex | 3.76E-05 | 1.53E-04 | 3.2% | 0.0% |
| proton-transporting two-sector ATPase complex | 1.22E-04 | 4.84E-04 | 4.3% | 0.2% |
| mitochondrial respiratory chain compex Ⅰ | 1.33E-04 | 4.92E-04 | 4.3% | 0.2% |
| respiratory chain complex Ⅰ | 1.33E-04 | 4.92E-04 | 4.3% | 0.2% |
| NADH dehydrogenase complex | 1.33E-04 | 4.92E-04 | 4.3% | 0.2% |
| mitochondrial proton-transporting ATP synthase complex | 1.61E-04 | 5.71E-04 | 3.2% | 0.1% |
| proton-transporting two-sector ATPase complex, catalytic domain | 1.61E-04 | 5.71E-04 | 3.2% | 0.1% |
| proton-transporting ATPase complex | 2.19E-04 | 7.60E-04 | 3.2% | 0.1% |
| small ribosomal subnit | 3.50E-04 | 1.19E-03 | 4.3% | 0.3% |
| mitochondrial proton-transporting ATP synthase complex, catalytic core F(1) | 4.72E-04 | 1.57E-03 | 2.1% | 0.0% |
| ribosomal subnit | 6.05E-04 | 1.97E-03 | 5.3% | 0.7% |
| proton-transporting ATP synthase complex, catalytic core F(1) | 6.58E-04 | 2.10E-03 | 2.1% | 0.0% |
| macromolecular complex | 9.42E-04 | 2.95E-03 | 33.3% | 19.3% |
| mitochondrial proton-transporting ATP synthase complex, catalytic core | 5.68E-03 | 1.72E-02 | 1.0% | 0.0% |
| proton-transporting ATP synthase synthase, catalytic core | 5.68E-03 | 1.72E-02 | 1.0% | 0.0% |
| citrate lyase complex | 1.13E-02 | 3.36E-02 | 1.0% | 0.0% |
| cell part | 1.61E-02 | 4.48E-02 | 97.8% | 91.8% |
| cell | 1.61E-02 | 4.48E-02 | 97.8% | 91.8% |
| non-membrane-bounded organelle | 1.62E-02 | 4.48E-02 | 23.6% | 14.8% |
| intracellular non-membrane-bounded organelle | 1.62E-02 | 4.48E-02 | 23.6% | 14.8% |
