# Supplementary material for: Mitochondrial metabolic study guided by proteomics analysis in hepatocellular carcinoma cells surviving long-term incubation with the highest dose of sorafenib
Source: Aging (Albany NY). 2019 Dec 26;11(24):12452–75. doi: 10.18632/aging.102582 (PMC6949094; doi:10.18632/aging.102582)
Supplement: Supplementary Table 3 [file aging-11-102582-s003..pdf]

Supplementary Table 3. Quantitative proteomic data for the proteins associated with mitochondrial oxidative phosphorylation expressed in the control, LI_{72h}, MI_{72h} and HI_{72h} HepG2 cells as related to Figure 2F.

Accession	Gene name	Control cells	LI_{72h} cells	MI_{72h} cells	HI_{72h} cells
O95169	NDUFB8	158.2083925	214.195318	206.9290562	291.7688383
O75489	NDUFS3	3892.410854	4183.375842	4432.450623	4456.458613
O00217	NDUFS8	1494.286484	2239.430863	2410.356302	3620.533131
E7EPT4	NDUFV2	3322.635741	3958.815089	4704.07201	4949.594508
P56556	NDUFA6	1880.422222	2797.318436	3332.465009	5094.896942
Q16718	NDUFA5	1815.287766	1880.829421	1996.174188	3473.09675
P21912	SDHB	5273.728415	5515.618183	7233.768921	9261.138678
P47985	UQCRFS1	3660.764012	3758.677005	4170.657287	4880.144213
P09669	COX6C	357.417757	378.7643425	482.3304618	686.3551529
P14854	COX6B1	979.8713339	1022.770219	1186.278055	2580.330664
H0YI37	ATP5B	332.5922739	499.1737755	544.3227782	1557.587768
P36542	ATP5C1	7636.128309	8857.689909	9350.255724	13224.4614
O75947	ATP5H	5531.32524	6259.657603	7875.508197	9605.480226
